# Early Detection of Cell Death Using Transmembrane Water Exchange Magnetic Resonance Imaging

**DOI:** 10.1002/advs.202513317

**Published:** 2025-12-17

**Authors:** Athanasia Kaika, Luca Nagel, Ulrike Höckendorf, Bangwen Xie, Geoffrey J. Topping, Mathias Schillmaier, Irina Beer, Frits H. A. van Heijster, Julian Rauch, Tristan A. Kuder, Sandra Sühnel, Bernd Erber, Christian Lohrmann, Simone Ballke, Thomas Metzler, Katja Steiger, Philipp J. Jost, Natalia P. Ivleva, Philipp Paprottka, Jonathan Nadjiri, Kevin M. Brindle, Wolfgang A. Weber, Franz Schilling

**Affiliations:** ^1^ Department of Nuclear Medicine TUM School of Medicine and Health TUM University Hospital Technical University of Munich Ismaninger Str. 22 81675 Munich Germany; ^2^ Department of Internal Medicine III TUM School of Medicine and Health TUM University Hospital Technical University of Munich Ismaninger Str. 22 81675 Munich Germany; ^3^ Cancer Research UK Cambridge Institute Li Ka Shing Centre University of Cambridge Robinson Way CB2 2RE Cambridge UK; ^4^ Chair of Analytical Chemistry and Water Chemistry Institute of Water Chemistry TUM School of Natural Sciences (NAT, Dep. Chemistry) Technical University of Munich Lichtenbergstr. 4 85748 Garching Germany; ^5^ Division of Medical Physics in Radiology German Cancer Research Center (DKFZ) Im Neuenheimer Feld 223 69120 Heidelberg Germany; ^6^ Comparative Experimental Pathology (CEP), Institute of Pathology TUM School of Medicine Technical University of Munich Trogerstr. 18 81675 Munich Germany; ^7^ German Cancer Consortium (DKTK) partner site Munich, a partnership between DFKZ and TUM Im Neuenheimer Feld 280 69120 Heidelberg Germany; ^8^ Department of Oncology University Clinic for Internal Medicine Medical University of Graz Auenbruggerplatz 15 8036 Graz Austria; ^9^ Department of Interventional Radiology TUM School of Medicine and Health Technical University of Munich Ismaninger Str. 22 81675 Munich Germany; ^10^ Department of Biochemistry University of Cambridge Tennis Court Road CB2 1QW Cambridge United Kingdom; ^11^ Institute for Advanced Study Technical University of Munich Lichtenbergstr. 2 a 85748 Garching Germany; ^12^ Bavarian Cancer Research Center (BZKF) 81675 Munich Germany; ^13^ Munich Institute of Biomedical Engineering Technical University of Munich Boltzmannstr. 11 85748 Garching Germany; ^14^ Faculty of Physics and Astronomy Heidelberg University Im Neuenheimer Feld 226 69120 Heidelberg Germany

**Keywords:** cell death, diffusion, MRI, transmembrane water exchange

## Abstract

Cell death plays a key role in cancer progression and treatment. After its onset, cell membrane integrity is often compromised. Here, it is shown in cells, animals and humans that the transmembrane water exchange rate, measured using magnetic resonance filter‐exchange spectroscopy (FEXSY) or imaging (FEXI), and quantified by the apparent exchange rate (AXR), is an early biomarker of cell death. AXR measurements detect the onset of cell death in vivo earlier than diffusion‐weighted MRI (DWI) and in vitro with similar sensitivity as flow cytometry. ^13^C magnetic resonance imaging (MRI) measurements of the malate/fumarate ratio (MFR), following injection of hyperpolarized [1,4‐^13^C_2_]fumarate, in tumors displaying diffuse necrosis, are correlated with AXR. AXR detects early treatment response in colorectal and lymphoma tumor models while the apparent diffusion coefficient (ADC) measured by DWI remains unchanged. AXR measurements in human uterine fibroids show sensitivity to post‐treatment changes soon after embolization, demonstrating clinical translatability of the method.

## Graphical abstract

Cell death is important in both the development and treatment of cancer. In this study, it is demonstrated for the first time that a specific measurement of the transmembrane water exchange rate using magnetic resonance imaging can be used as an early marker of cell death in mammalian cells, in animals and in human patients.

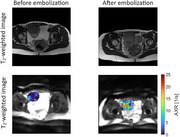

## 1 Introduction

Despite improvements in surgery, radiation, and targeted therapies, the clinical success of many anti‐cancer strategies remains disappointing because the therapy is usually not tailored to the individual patient's tumor biology.^[^ ^1^ ^]^ Treatment plans require a better understanding of the relationship between individual tumor biology and therapeutic approach to improve the patients’ outcome. The need for novel technologies to progress from late‐stage to early‐stage detection and to match patients with the most promising therapeutic strategy is considered essential for the concept of precision medicine, both for cancer and for many other diseases.^[^ ^2^ ^]^

Imaging techniques, including magnetic resonance imaging (MRI), are widely used for treatment response evaluations that are based on changes in tumor size^[^ ^3^ ^]^ and delineation, as well as signal enhancement after systemic contrast agent injection. However, before morphological and size changes become manifest, a successful treatment may first alter tumor cell viability.^[^ ^4^ ^]^ For the detection of treatment response, functional imaging techniques are used clinically to extract information about tumor metabolism, physiology, and cellular composition.^[^ ^5^ ^]^ These include diffusion‐weighted imaging (DWI), which allows the calculation of the apparent diffusion coefficient (ADC), that is sensitive to the underlying microstructural pathology and has been negatively correlated with tumor cell density.^[^ ^6^ ^]^ Although an increase in tumor ADC is associated with successful treatment, it occurs several days after the treatment that initiates cell death.^[^ ^7^, ^8^ ^]^ Both cell loss and loss of plasma membrane integrity contribute to the increase in ADC. However, due to the late response of ADC to treatment, the conventional DWI signal appears to be governed principally by cell loss rather than a loss of plasma membrane integrity, which happens earlier after the onset of cell death.^[^ ^9^ ^]^

An increase in plasma membrane water permeability was classically considered to be a hallmark of necrotic cells, whereas plasma membrane integrity was thought to be preserved during programmed cell death or apoptosis. Recent studies, however, have revealed that other forms of programmed cell death exist, including programmed necrosis,^[^ ^10^ ^]^ necroptosis,^[^ ^11^, ^12^, ^13^ ^]^ and pyroptosis,^[^ ^14^, ^15^, ^16^ ^]^ which involve translocation of channel or pore proteins to the plasma membrane. These proteins increase plasma membrane water permeability changes, leading finally to plasma membrane rupture. In addition, secondary necrosis, which was previously believed to be a non‐regulated form of cell lysis that occurred after apoptosis, can also be programmed, leading to a progressive loss of plasma membrane integrity in apoptotic cells.^[^ ^17^, ^18^ ^]^ Therefore, increased plasma membrane water permeability appears to represent a near universal early biomarker of cell death.

Diffusion exchange spectroscopy (DEXSY) allows the estimation of water exchange between the compartments of a biological system.^[^ ^19^, ^20^ ^]^ DEXSY has been used for ex vivo neonatal mouse spinal cords.^[^ ^21^ ^]^ However, the long acquisition time and the complex signal analysis limit its applications. Those limitations are overcome by filter‐exchange spectroscopy (FEXSY) introduced later by Aslund et al.^[^ ^22^ ^]^

Filter‐exchange spectroscopy (FEXSY) and imaging (FEXI) have been proposed as non‐invasive MR techniques for measuring intercompartmental water exchange, which may be related to cell membrane water permeability when applied to a biological system like cell suspensions or tissue.^[^ ^19^, ^22^, ^23^, ^24^, ^25^, ^26^, ^27^ ^]^ FEXSY and FEXI exploit the differences in diffusivity between intra‐ and extracellular water, resulting from the presence of the plasma membrane and intracellular structures. Using the filter‐exchange MR signal, the apparent exchange rate (AXR) can be calculated to give a quantitative assessment of water exchange across the plasma membrane and therefore, could provide an early imaging biomarker of treatment response.^[^ ^24^, ^28^ ^]^ Additionally, tumor necrosis has been proposed as a potential prognostic and staging factor that has been linked with tumor aggressiveness^[^ ^29^, ^30^ ^]^ and therefore, it may also be possible to use AXR as a diagnostic indicator (Figure S1, Supporting Information).

As an alternative clinical marker of cell death following treatment response, ^18^F‐labeled 2‐fluoro‐2‐deoxy‐D‐glucose (FDG) positron emission tomography/computed tomography (PET/CT) is now commonly used clinically, especially in lymphomas.^[^ ^4^, ^31^ ^]^ However, it reports on glucose uptake rather than early cell death. Therefore, the loss of the FDG signal from tumor cells following effective therapy can be confounded by FDG uptake by inflammatory cells and repair processes occurring after effective therapy. In addition, a restricting factor for PET is the associated patient exposure to ionizing radiation, which limits the number of follow‐up examinations or its use as a screening tool. Tumor cell death following treatment can also be assessed using ^13^C MRI and hyperpolarized (HP) [1,4‐^13^C_2_]fumarate.^[^ ^32^ ^]^ Following injection into lymphoma‐bearing mice, there was an increase in the concentration of labeled malate following etoposide treatment, which was attributed to a loss of plasma membrane integrity, allowing more rapid access of the enzyme fumarase to the injected fumarate. Recently, deuterium magnetic resonance measurements utilizing malate production from injection or oral ingestion of ^2^H‐labeled fumarate have been shown to provide an alternative and more sensitive method for detecting tumor cell death following treatment.^[^ ^33^, ^34^, ^35^ ^]^ Imaging with hyperpolarized ^13^C‐labeled fumarate or ^2^H‐labeled fumarate has also been shown to be more sensitive for detecting low levels of diffuse necrosis than DWI.^[^ ^35^, ^36^ ^]^

We showed recently that the AXR is sensitive to yeast cell membrane alterations that take place during the early stages of necrosis.^[^ ^37^ ^]^ We show here that the AXR can be used as an early marker of multiple forms of cell death in vitro in human leukemia cells. We demonstrate the use of AXR as an imaging biomarker to detect early evidence of treatment response in colorectal and lymphoma animal tumor models and in patients following uterine fibroid embolization.

## 2 Results

### 2.1 AXR Detection of Cell Death In Vitro

**Figure 1:**
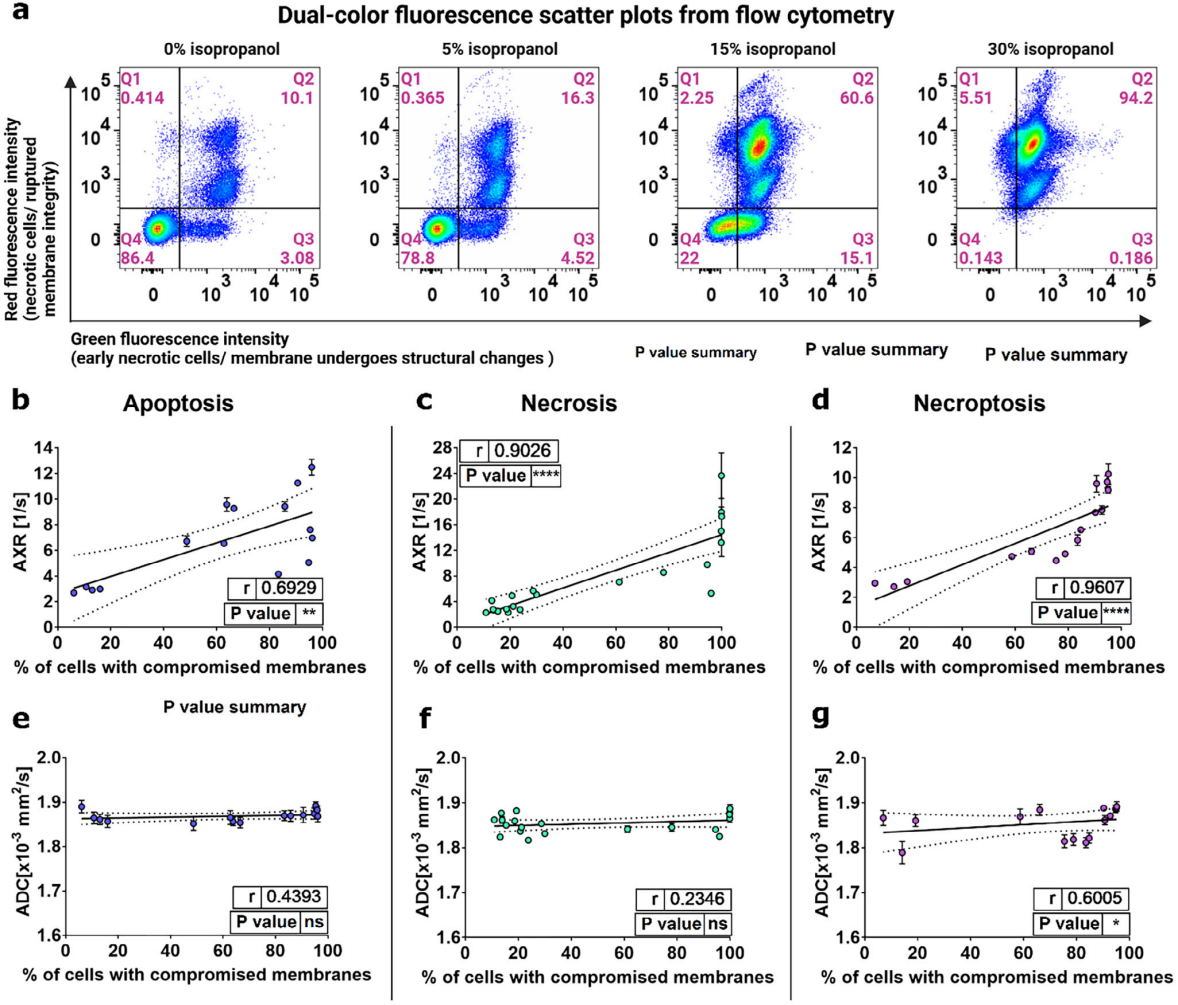
Flow cytometry, FEXSY, and pulsed gradient spin echo (PGSE) measurements on AML cells induced to undergo apoptosis, necrosis, and necroptosis by treatment with varying concentrations of doxorubicin, isopropanol, and emricasan/birinapant/tumor necrosis factor (TNF), respectively. a), Representative flow‐cytometric plots of AML cells treated with isopropanol after staining with Annexin V and propidium iodide (PI). The cell population of the individual gates represents the percentage of: live cells (Q4, Annexin V(‐)/PI(‐)); early necrotic cells (Q3, Annexin V(+)/PI(‐)); late necrotic cells (Q2 + Q1, Annexin V(+)/PI(+) + Annexin V(‐)/PI(+)). b–d), AXR and e–g) ADC plotted as a function of the percentage of cells with compromised plasma membranes. Shown are the mean and standard deviation of three measurements on the same sample. Cells labeled as “cells with compromised membranes” are considered cells with plasma membranes undergoing structural changes (Annexin V(+)) and/or with loss of integrity (PI(+)). Those cells appeared in the gates Q1, Q2, and Q3 of the dual‐color fluorescence scatter plots from flow cytometry. Correlation coefficients (r) and *p*‐values were calculated using Spearman correlation; n.s. *p* > 0.05, ^*^ *p* ≤ 0.05, ^**^ *p* ≤ 0.01, ^***^ *p* ≤ 0.001, ^****^ *p* ≤ 0.0001.

For apoptotic, necrotic, and necroptotic death of human acute myeloid leukemia (AML) cells, there was a positive correlation between AXR values and the fraction of cells with compromised membranes (stained positively by Annexin V and/or propidium iodide (PI)) detected with flow cytometry (**Figure** **1**a–d; Figure S2, Supporting Information). This is in contrast to measurements of the ADC, which showed no significant increase for cells undergoing apoptosis or necrosis (Figure 1e,f; Figure S2, Supporting Information) and only a small, but significant, increase for cells undergoing necroptosis (Figure 1g). **Table** **1** summarizes AXR values, ADC values, and the cell fractions with ruptured plasma membranes (stained positively by PI), as determined by flow cytometry (see also Figure S2, Supporting Information).

**Table 1:** Stimulus concentration or percentage and the corresponding mean ± standard deviations of AXRs, ADCs (calculated from DW‐MRS signal) and fractions of AML cells with ruptured membrane (sum of Annexin V (‐)/ PI (+) and Annexin V (+)/ PI (+)) measured by flow cytometry.

Stimulus [unit of concentration]	Stimulus concentration	AXR [1/s]	ADC [×10^−3^ mm^2^ s^−1^]	Cells with ruptured membrane [%]
Doxorubicin [µm]	0	3.0 ± 0.2	1.87 ± 0.002	8 ± 5
	0.1	4.5 ± 1.8	1.865 ± 0.004	44 ± 36
	0.5	7 ± 2	1.865 ± 0.012	67 ± 34
	1	9.4 ± 1.8	1.872 ± 0.019	68 ± 26
	2.5	10 ± 3	1.869 ± 0.013	66 ± 28
Birinapant [nm]	0	2.9 ± 0.2	1.84 ± 0.04	7 ± 3
	10	5.7 ± 1.7	1.86 ± 0.04	37 ± 15
	100	6.3 ± 2.5	1.86 ± 0.04	48 ± 20
	250	8.6 ± 2.4	1.86 ± 0.04	56 ± 11
	1000	8.0 ± 1.5	1.85 ± 0.03	59 ± 5
Isopropanol [volume %]	0	2.6 ± 0.3	1.87 ± 0.001	10.1 ± 1.3
	3	2.6 ± 0.3	1.86 ± 0.02	13.3 ± 1.3
	5	3.4 ± 0.7	1.83 ± 0.01	15 ± 5
	10	5.3 ± 0.4	1.84 ± 0.01	21 ± 6
	15	7.0 ± 1.6	1.84 ± 0.01	59 ± 24
	20	13 ± 3	1.86 ± 0.02	80 ± 31
	30	20 ± 3	1.88 ± 0.01	99.1 ± 0.6

**Figure 2:**
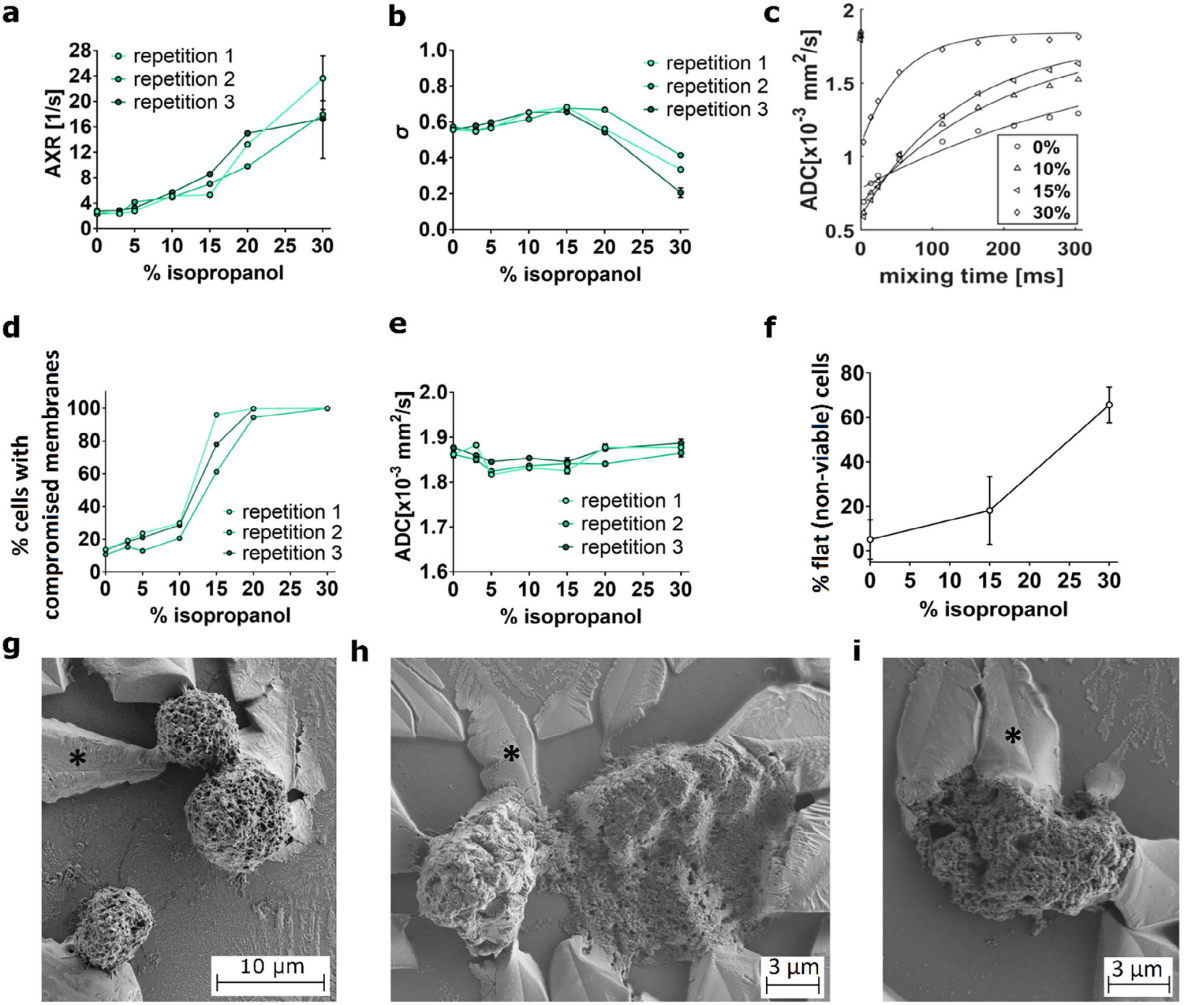
FEXSY, flow cytometry, PGSE, and SEM data from AML cells induced to undergo necrosis by treatment with isopropanol. a), Individual AXR measurements and b), filter efficiency (σ) as a function of the isopropanol concentrations. c), AXR relaxation curves. d), Measurements of the percentages of cells with compromised membranes (percentage of cells in the gates Q1 + Q2 + Q3) and e), individual ADC measurements as a function of the isopropanol concentrations. f), Percentage of flat non‐viable cells in SEM images. SEM images of g) untreated cells, h) cells treated with 10% isopropanol and i) cells treated with 30% of isopropanol. ^*^ PBS precipitate crystals.

For cells induced to undergo necrosis with isopropanol, in general increasing isopropanol concentration led to an increase in the percentage of cells with compromised membranes, while the AXR increased gradually and the ADC remained constant (**Figure** **2**). Concentrations above 15% led to a loss of filter efficiency in the FEXSY experiment (Figure 2b), and at 30% isopropanol the AXR fits were less robust leading to high variability in the individual AXR measurements (Figures 1c and 2a).

For 0% of isopropanol, only 10% of cells had ruptured membranes and AXR was equal to 2.6 1/s. For 30% of isopropanol, almost the entire cell population was necrotic (Figure 2d) and the AXR increased to 20 1/s (Table 1; Figure S2, Supporting Information). Control and isopropanol‐treated cells were imaged with scanning electron microscopy (SEM) to investigate the external morphological cell features induced by isopropanol. Concentrations of more than 15% of isopropanol caused cells to lose their spherical configuration (Figure 2f–i), a previously described consequence of cell membrane breakdown.^[^ ^38^ ^]^

### 2.2 Detecting Tumor Cell Death In Vivo Using AXR Measurements

**Figure 3:**
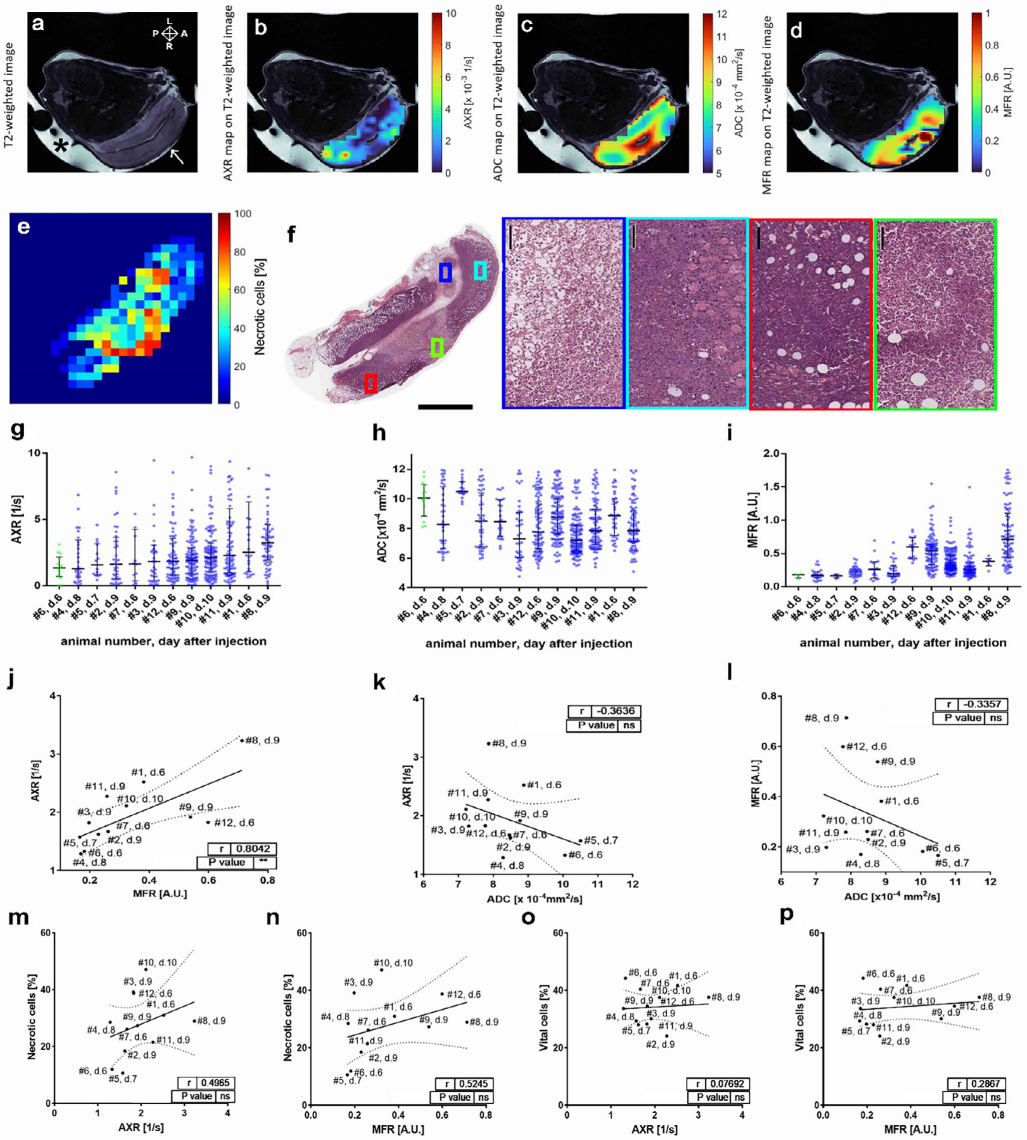
FEXI, hyperpolarized ^13^C and diffusion‐weighted images of EL4 lymphoma tumors. Tumors developed and were imaged 6, 7, 8, and 9 days after the subcutaneously injection of EL4 lymphoma tumor cells (ATCC) on the lower back of female mice. a), Representative axial T_2_‐weighted ^1^H image (^*^ indicates the gel used to improve B0 shim uniformity and the white arrow points to the tumor). The image orientation is demonstrated on the top right where A: anterior, P: posterior, R: right, L: left. b), AXR, c), ADC) and d), hyperpolarized ^13^C MFR maps of the solid tumor regions (tumor regions with ADC higher than 12 × 10^−4^ mm^2^ s^−1^ have been excluded) overlaid on a T_2_‐weighted image. e), Heat map of the percentage of necrotic cells determined from the H&E stained image shown in f). In the left‐most panel of (f), the scale bar = 3 mm. In the other panels of (f), he scale bar = 100 µm (with colored representative regions shown in high magnification). g), AXR, h), ADC and i), MFR scatter plots for the 12 tumor‐bearing animals examined. Each data point represents a tumor voxel value. The horizontal lines correspond to the median values and the whiskers range to the 95% confidence interval for the median values. The voxel values colored in green correspond to an animal that we chose as a reference for low levels of observed necrosis (low AXR and low standard deviation of the AXR in combination with histology, see panel (m). j–l), Correlation plots between the median AXR, MFR and ADC voxel values. m–p), Correlation plots between tumors’ median AXR, MFR voxel values and the mean percentages of necrotic and viable cells. Solid and dotted lines represent the linear regression line and the 95% confidence bands of the best‐fit line, respectively. Each point is labeled with the corresponding animal number (#) and the number of days after tumor cell injection (d.). Correlation coefficients (r) and *p*‐values were calculated using Spearman correlation; n.s., *p* > 0.05, ^*^ *p* ≤ 0.05, ^**^ *p* ≤ 0.01.

Representative MR images of mice with untreated subcutaneously implanted murine lymphomas (EL4) are shown in **Figure** **3**. Tumor AXR voxel values ranged from 0.01 to 33.34 1/s, but only 1.1% were higher than 20 1/s. The AXR filter efficiency (σ) ranged between 0.02 and 0.97. The ADC voxel values ranged from 4.7 to 12.0 × 10^−4^ mm^2^ s^−1^. The malate/fumarate voxel values (MFR) in animals injected with hyperpolarized [1,4‐^13^C_2_]fumarate were between 0.04 and 1.8, with only 2% higher than 1.3 (**Table** **2**). The interquartile range (IQR) and median voxel values indicated a greater variation in AXR and MFR than in ADC.

**Table 2:** FEXI, DWI and CSI results of EL4 lymphomas and uterine fibroids before and after embolization. The median values, the 95% range and the IQR/Median were calculated, including all the voxel values of all solid tumor regions.

		AXR [1/s]	σ	ADC [mm^2^ s^−1^]	MFR
EL4 lymphoma	Median	1.87	0.4	8.1 ×10^−4^	0.28
	95% Range	0.11–10.75	0.17–0.70	(5.8 –11.3) ×10^−4^	0.07–0.99
Uterine fibroids before embolization	Median	0.9	0.3	1.6 ×10^−3^	–
	95% Range	0.07–5.12	0.17–0.48	(1.24 –1.98) x10^−3^	–
Uterine fibroids after embolization	Median	5.1	0.2	1.0 ×10^−3^	–
	95% Range	0.55 – 28.59	0.06 – 0.53	(0.82–1.27) ×10^−3^	–
		AXR	σ	ADC	MFR
EL4 lymphoma	IQR/Median	1.4	0.5	0.3	1.0

Histological analysis of EL4 tumor slices showed regions occupied by tumor cells, edema and hemorrhage (Figure 3f). The tumor cells infiltrated the skin as well as the adjacent skeletal muscle and adipose tissue. The tumors mainly showed diffuse necrosis containing variable amounts of necrotic, viable, and mitotic tumor cells as well as confluent areas of only necrotic cells.

Median AXR values showed a statistically significant positive correlation with median MFR values (Figure 3j) but no correlation with median ADC values (Figure 3k). Furthermore, there was no correlation between MFR and ADC values (Figure 3l). There were weak positive correlations between the percentage of necrotic cells and AXR and MFR, which were not significant (Figure 3m,n), but no correlations between the percentage of viable cells and AXR and MFR (Figure 3o,p).

### 2.3 AXR as a Marker of Early Treatment Response in Two In Vivo Tumor Models

The effects of treatment induced cell death on MR signals were studied for two mechanistically different forms of therapy in a lymphoma and a colorectal cancer model. Lymphomas were treated with etoposide, a small‐molecule chemotherapy drug that significantly alters tumor proteins, resulting in apoptosis.^[^ ^39^ ^]^ The colorectal cancer model was treated with agonist MEDI3039 that triggers apoptotic cell death after attaching to TRAILR2 receptors.^[^ ^40^ ^]^

**Figure 4:**
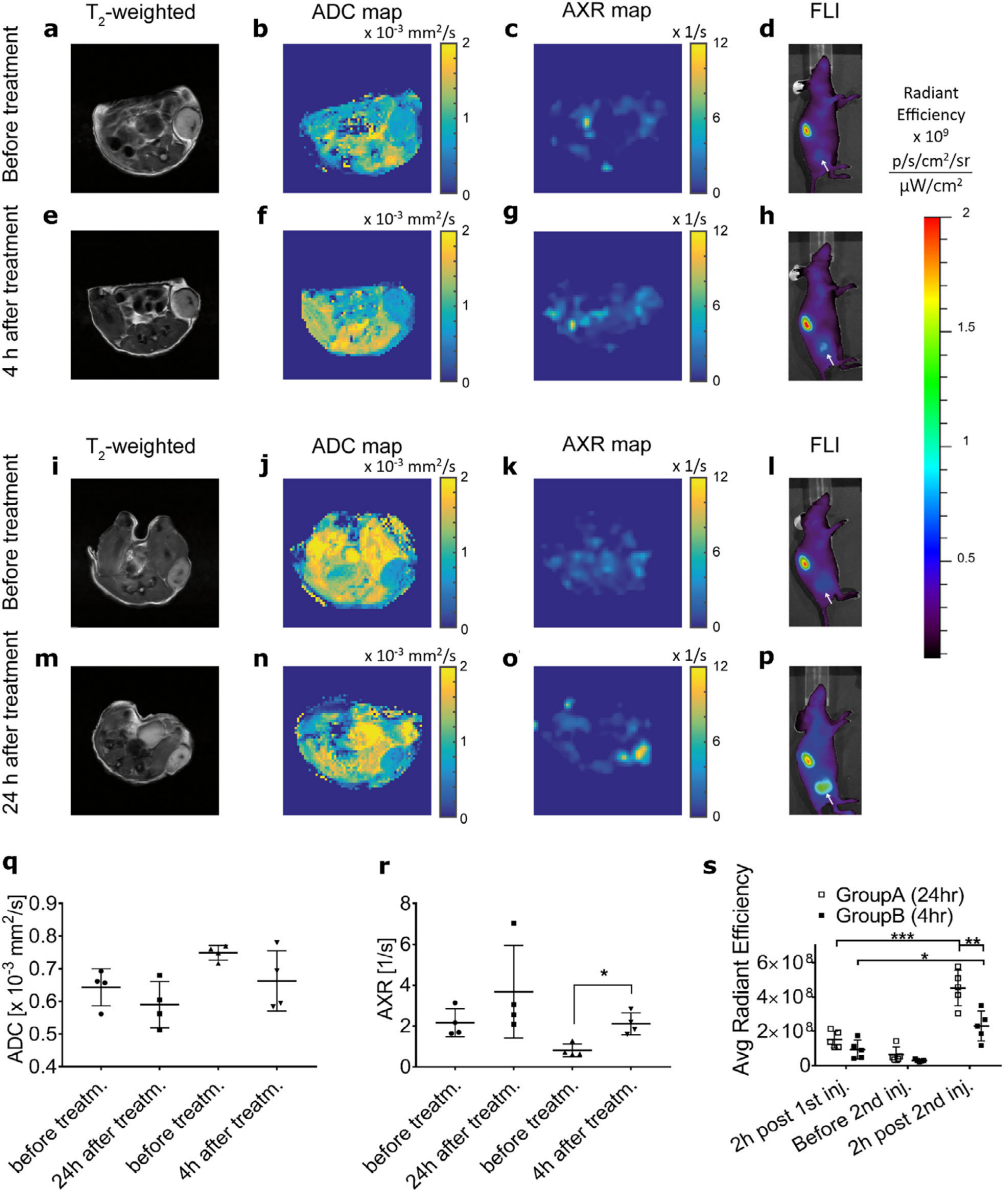
Detection of cell death in colorectal tumor xenografts treated with a TRAIL R2 agonist. T_2_‐weighted a,e,i,m), diffusion‐weighted b,f,j,n), and FEXI c,g,k,o) MR images and NIR fluorescence images of animals injected with C2Am‐AF750 d,h,l,p) were acquired before and at 4 and 24 h after treatment with MEDI3039. The white arrow in fluorescence images points to the tumor. ADC q), AXR r) and average radiant efficiency s) of the colorectal tumor xenografts imaged before and 4 or 24 h after treatment. The first injection of C2Am‐AF750 was done 24h before treatment and fluorescence imaging was performed twice (24 h and directly before treatment), whereas the second injection of C2Am‐750 and third fluorescence imaging were performed 2 h post‐treatment. In the scatter plots, each point represents the mean value for one tumor and the horizontal lines and whiskers correspond to the mean and standard deviations for five tumors. A statistical analysis was conducted using two‐tailed, pairwise Student's *t*‐tests, ^*^ *p* ≤ 0.05, ^**^ *p* ≤ 0.01, ^***^ *p* ≤ 0.001.

In the colorectal cancer model, subcutaneous Colo205 tumors were treated with the TRAIL receptor agonist, MEDI3039. There was no change in ADC following treatment (**Figure** **4**q) but there was a significant increase in AXR at 4 h post‐treatment, but not at 24 h (Figure 4r). An increase in cell death following treatment was confirmed in vivo by NIR fluorescence detection of a fluorescently labeled peptide (C2Am‐AF750) that binds to both apoptotic and necrotic cells (Figure 4s). An increase in cell death post‐treatment was confirmed by staining tumor sections with C2Am‐AF750 and an antibody for cleaved caspase 3 (Figure S5, Supporting Information).

The same measurements were made in subcutaneous EL4 lymphoma tumors following treatment with etoposide (Figure S6, Supporting Information). There were no changes in ADC following treatment (Figure S6q, Supporting Information), but there was a significant increase in AXR at 24 h post‐treatment, but not at 6 h (Figure S6r, Supporting Information). There was also a significant increase in the tumor concentration of C2Am‐AF750 at 24 post‐treatment but not at 6 h (Figure S6s, Supporting Information). Again, an increase in cell death post‐treatment was confirmed by staining tumor sections with C2Am‐AF750 and an antibody for cleaved caspase 3 (Figure S7, Supporting Information).

### 2.4 AXR as a Marker of Early Treatment Response in the Clinic

**Figure 5:**
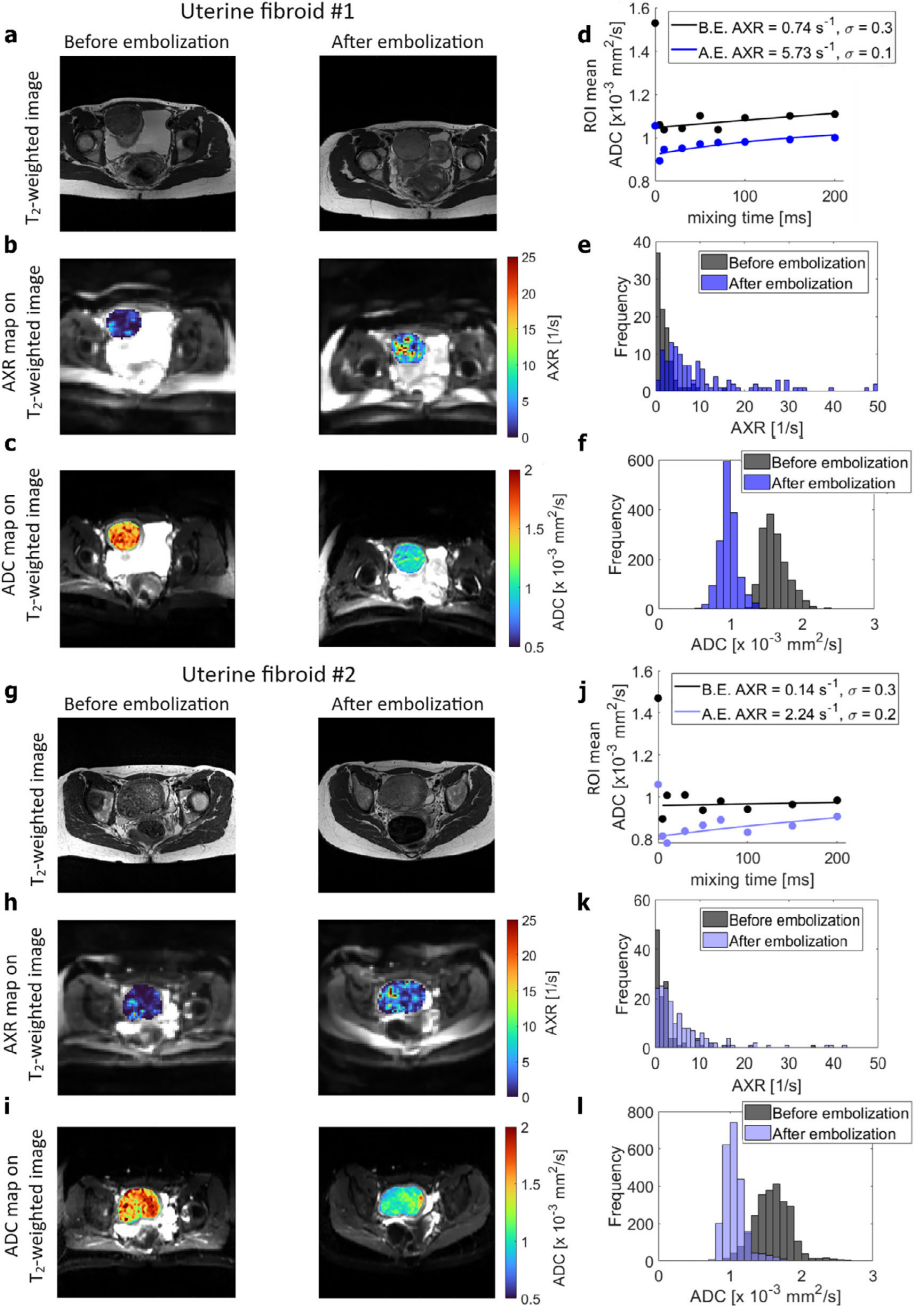
Detection of treatment response in two patients with uterine fibroids. T_2_‐weighted images a,g). AXR b,h) and ADC maps c,i), overlaid on T_2_‐weighted images, of uterine fibroids before and 24 h after embolization. d,j) AXR relaxation curves, before and after embolization (B.E., A.E.), with AXR fit calculated from the mean ADC values selected from a region‐of‐interest covering the entire uterine fibroid illustrated in b and h, respectively. AXR value histograms e,k) of uterine fibroids illustrated in b and h, respectively. ADC value histograms f,l) of uterine fibroids are illustrated in c and i, respectively.

AXR and ADC maps were acquired from uterine fibroids in two patients before and 24 h after embolization treatment (**Figure** **5**). The ADC values were similar to those found in a previous study on uterine fibroids.^[^ ^41^ ^]^ The AXR values before and after embolization are within the range found in other tissues and in tumors.^[^ ^24^, ^25^, ^28^, ^42^ ^]^ Following treatment median AXR increased, whereas the filter efficiency and ADC decreased (Table 2). Moreover, the distribution of AXR values was found to be broader after embolization (Figure 5e,k).

## 3 Discussion

Apoptosis, necrosis, and necroptosis cell death pathways involve changes in plasma membrane composition and water permeability. Here, we induced these cell death pathways in AML cells and confirmed their induction by using flow cytometry to measure the exposure of phosphatidylserine on the surface of apoptotic cells and an increase in plasma membrane water permeability in necrotic cells. By increasing the concentrations of the agents that induced these pathways, we produced cell populations that shifted from viable cells with intact membranes to apoptotic/early necrotic cells with plasma membranes undergoing structural rearrangements to late necrotic cells with permeabilized plasma membranes.

PGSE measurements of the ADC of water showed that this did not change as the fraction of cells with disrupted plasma membranes increased. This is in contrast to the results of studies on solid tumors where effective treatments can result in an increase in ADC. This occurs as a result of a loss of tumor cellularity^[^ ^6^ ^]^ caused by phagocytic clearance mechanisms,^[^ ^43^ ^]^ a mechanism that is absent from these in vitro experiments. FEXSY measurements, however, showed an increase in transmembrane water exchange (AXR) as the fraction of cells with a compromised plasma membrane increased, establishing AXR as a biomarker for cell death in vitro. The repeatability and reproducibility of FEXI‐based AXR measurements have been established previously in measurements on yeast cells^[^ ^26^ ^]^ and here in vivo from measurements on a subcutaneous lymphoma model.

The timing of the initialization of membrane permeabilization during cell death is affected by a number of factors including the type of cell death and the cells’ microenvironment.^[^ ^44^, ^45^ ^]^ During apoptotic cell death, which is often induced by cancer treatment,^[^ ^46^, ^47^ ^]^ phagocytes engulf dying cells and prevent the progression of apoptosis to secondary necrosis.^[^ ^45^ ^]^ However, upon extensive apoptosis, where the rate of apoptotic events exceeds the phagocytic capacity of the tissue, secondary necrosis results, which includes permeabilization of the apoptotic cell plasma membrane.^[^ ^48^, ^49^ ^]^ Such secondary necrosis can occur following tumor chemotherapy and radiotherapy.^[^ ^10^ ^]^ Upon necroptosis, another form of cell death induced by cancer treatment,^[^ ^50^ ^]^ cell membrane alterations are an early event, when compared to apoptosis, and are linked to the immune response.^[^ ^10^, ^51^, ^52^ ^]^ Therefore, in principle, measurements of transmembrane water exchange (AXR) may provide an earlier indication of cell death than ADC measurements of a loss of tumor cellularity.

The in vivo measurements of ADC and AXR in EL4 lymphomas were in the same range as those measured in other tumors and tissues.^[^ ^24^, ^25^, ^28^, ^42^, ^53^, ^54^ ^]^ High ADC contrast was observed between regions with different cell densities, consistent with previous studies.^[^ ^7^, ^55^ ^]^ ADC was relatively uniform in solid tumor regions when compared to AXR and the MFR in animals injected with hyperpolarized [1,4‐^13^C_2_]fumarate. There was a significant positive correlation between AXR and MFR consistent with FEXI measuring increased transmembrane water exchange in necrotic cells, although a direct correlation between AXR and spontaneous necrosis assessed histologically was not demonstrated in this study.

In Colo205 xenografts treated with a TRAIL R2 agonist AXR was increased at 4 h post‐treatment but not at 24 h. ADC showed no effect of treatment up to 24 h post‐treatment. A different pattern was observed in mice bearing EL4 lymphomas that were treated with etoposide. AXR and C2Am‐AF750 indicated the presence of dead cells at 24 h after treatment but not at 6 h, while ADC remained unchanged. These results are consistent with our previous study in yeast cells where AXR detected membrane alterations that appeared during the early stages of cell death but failed to detect membrane breakdown when there was extensive necrosis resulting in a loss of transmembrane water exchange.^[^ ^36^ ^]^

Clinical studies in two patients with uterine fibroids demonstrated the clinical translatability of the AXR measurements for detecting cell death. Acute vascular occlusion caused by embolization leads to a condition comparable to ischemic stroke. After a stroke, the ADC initially decreases and then later increases.^[^ ^56^ ^]^ The initial ADC decrease has been attributed to cell swelling, which is followed by progressive membrane permeabilization, and the late ADC increase has been attributed to necrosis, including extensive membrane disruption.^[^ ^9^, ^57^, ^58^, ^59^, ^60^ ^]^ Cell swelling and membrane permeabilization before necrosis are possible explanations for the observed reduction in uterine fibroid ADC and increase in AXR following embolization.

The advantage of FEXI compared to other alternative methods for cell death detection, such as hyperpolarized fumarate^[^ ^32^ ^]^ and deuterated fumarate,^[^ ^33^ ^]^ is that FEXI does not require an exogenous contrast agent or specialized equipment for X‐nuclei, facilitating the inclusion of FEXI in standard clinical protocols.

Increases in membrane water permeability are a relatively early event following treatment induced cell death which have little effect on ADC but can produce a marked increase in AXR. FEXI measurements of AXR can therefore provide an earlier indication of treatment induced cell death and the response of tumors to treatment.

## 4 Experimental Section

### Filter‐Exchange Sequence

The signal preparation of filter‐exchange spectroscopy (FEXSY) and imaging (FEXI) is identical, and only the detection module differs. FEXSY/FEXI employs two PGSE blocks separated by a varying time interval, named mixing time (t_m_). The gradients of the first PGSE block, or diffusion filter block, are set high enough to dephase the fast‐diffusing spins of the extracellular space. The gradients of the second PGSE block, which is referred to as the detection block, are incremented to measure the diffusivity of the spins that were not substantially attenuated by the diffusion filter, mainly those of the intracellular space during the filter application. Therefore, the ADC, or ADC_filter,on_ (t_m_ = 0), measured directly after the diffusion filter application, is lower compared to the ADC measured without the filter application. This reduction is described by the filter efficiency, which is defined:

(1) $$\sigma =1-\mathrm{AD}C_{\mathrm{filter},\mathrm{on}}\left(t_{m}=0\right)/\mathrm{AD}C_{\mathrm{filter},\mathrm{off}}$$

When the time interval following the diffusion filter, t_m_, increases, the initially intracellular molecules exchange to the extracellular space by crossing the cell membrane and then appear in the extracellular space, resulting in higher detected diffusivity (Figure S1, Supporting Information). When the t_m_ is long enough to allow exchange that results in equal intra‐ and extracellular fractional populations to the ones before the filter application, the measured ADC’ tends to be equal to the ADC before the filter application, and the filter influence has been eliminated. The above described exchange process is modeled by:

(2) $$\mathrm{AD}C_{\mathrm{filter},\mathrm{on}}\left(t_{m}\right)=\mathrm{AD}C_{\mathrm{filter},\mathrm{off}}\left(1-\sigma e^{-t_{m}\text{AXR}}\right)$$

where AXR is the apparent exchange rate and the ADC_filter on/off_ are determined by fitting the FEXSY/FEXI signal acquired for different b‐values of the detection block to the Stejskal–Tanner equation:

(3) $$S_{\mathrm{filter}\;\mathrm{on}/\mathrm{off}}\left(b\right)=S_{\mathrm{filter}\;\mathrm{on}/\mathrm{off},0}e^{-{\mathrm{bADC}}_{\mathrm{filter}\mathrm{on}/\mathrm{off}}}\;$$

### Cell Culture

The human AML cell line MOLM13 (RRID:CVCL_2119) was purchased from DSMZ (Leibniz, Germany) and maintained in Iscove's modified Dulbecco's medium (Gibco, Grand Island, USA) supplemented with 20% FCS (Pan Biotech, Aidenbach, Germany), 0.05 mm 2‐mercaptoethanol (Gibco, Grand Island, USA), and 0.05 mm penicillin/streptomycin (Gibco, Grand Island, USA) at 37 °C and CO_2_ with saturating humidity. Cells were regularly tested for mycoplasma using the MycoProbe Mycoplasma Detection Kit (R&D Systems, Minneapolis, USA), and authenticated utilizing Short Tandem Repeat (STR) profiling (ATCC). Mouse T lymphoma EL4 cells (ATCC Cat# TIB‐39, RRID:CVCL_0255) were cultured in formulated Dulbecco's Modified Eagle's Medium (Life Technologies Inc., Carlsbad, CA). Human colorectal adenocarcinoma Colo205 cells (ATCC, RRID:CVCL_F402)) were cultured in RPMI‐1640 medium (Life Technologies Inc.). Both media were supplemented with 10% fetal bovine serum (FBS; Lonza, Basel Switzerland). All cell lines were cultured in a humidified incubator at 37° C and 5% CO_2_ and were tested for mycoplasma with negative result.

### Induction of Cell Death In Vitro

50 × 10^6^ cells were treated with either doxorubicin, isopropanol or emricasan/birinapant/TNF to induce forced apoptosis, necrosis, and necroptosis, respectively. For induction of apoptotic cell death, cells were treated with 0, 0.1, 0.5, 1.0, or 2.5 µm of the standard chemotherapeutic doxorubicin (in‐house‐pharmacy) for 41 h under maintenance culture conditions.^[^ ^61^ ^]^ For induction of necrotic cell death, cells were resuspended in 2 mL Dulbecco's phosphate‐buffered saline (PBS) from Gibco (Grand Island, USA) and treated with 0, 3, 5, 10, 15, 20, or 30% isopropanol (v/v) (Merck KGaA, Darmstadt, Germany) for 5 min at room temperature (RT).^[^ ^62^ ^]^ For induction of necroptotic cell death, cells were pre‐treated for 30 min with 5 µm of the pan‐caspase inhibitor emricasan (Selleckchem, Houston, USA), followed by treatment with 0, 10, 100, 250, or 1000 nm of the second mitochondrial‐derived activator of caspases (SMAC) mimetic birinapant (Selleckchem, Houston, USA) and 100 ng mL^−1^ recombinant human (rh‐)TNF (Biolegend, California, USA) for 16 h under maintenance culture conditions.^[^ ^63^ ^]^

After treatment, cells were centrifuged (400 g, 5 min, RT), the supernatant was removed and the cell pellet resuspended in 2 mL PBS. For MR measurements, the cell suspension was transferred into 10 mm diameter glass NMR tubes (Merck KGaA, Darmstadt, Germany). 50 µL of each sample solution was used for flow cytometry, performed in parallel to the MR scan. Scanning electron microscopy required separately‐prepared samples, prepared according to the same protocols as described above.

### Mice and Tumor Models Without Therapy

Female C57BL/6 mice (N = 12, body mass = 19.6 ± 1.2 g, 7–8 weeks old, Charles River) were injected subcutaneously with one dose of 5 × 10^5^ EL4 lymphoma tumor cells (ATCC) in 100 µL PBS on the lower back and imaged with MRI at 6, 7, 8, and 9 days post‐injection. During imaging, mice were kept under isoflurane anesthesia (in 100% oxygen at flow rate 2 l/min, 5% by volume during induction and 1–3% by volume during scanning). A tail vein catheter was inserted while mice were under anesthesia and before being placed into the coil, and was used for delivery of the hyperpolarized agent while imaging. 240 µL of solution containing hyperpolarized agent at a concentration of 40 mm in 80 mm TRIS buffer (pH 7.5+/−0.2) was injected before the start of the CSI acquisition. All animal experiments with this group of mice were executed according to relevant laws and regulations and were approved by an ethical review board (Regierung von Oberbayern, ROB‐55.2‐2532.Vet_02‐17‐177, and related amendments).

### Mice and Tumor Models with Therapy

Female nude mice (BALB/c nu/nu) (N = 10, body mass ca. 20 g, 7–8 weeks old, Charles River Laboratories) bearing EL4 lymphoma tumors (implanted as described before) were treated with 70 mg kg^−1^ i.p. of etoposide two weeks after implantation and the other group received PBS as negative control.^[^ ^64^ ^]^ Before treatment and after another 6 h or 24 h, the mice were injected i.v. with 0.1 µmole kg^−1^ C2Am‐750. Female nude mice (N = 10, body mass ca 20 g, 7–8 weeks old, Charles River) bearing Colo205 colorectal tumors (implanted as described before) were treated with the TRAILR2 agonist MEDI3039 at 0.4 mg kg^−1^ (i.v.) and the other group received PBS as negative control.^[^ ^64^ ^]^ Before treatment and after 4 or 24 h, the mice were injected i.v. with 0.1 µmole kg^−1^ C2Am‐750. All animal experiments with this group of mice were performed in compliance with a project license issued under the Animals (Scientific Procedures) Act of 1986 and were designed with reference to guidelines of the U.K. Co‐ordinating Committee on Cancer Research for the welfare of animals in experimental neoplasia. Protocols were approved by the Cancer Research U.K., Cambridge Institute Animal Welfare and Ethical Review Body.

### Human Patients

Two clinical patients with uterine fibroids were scanned directly before and 24 h after the uterine fibroid embolization procedure. The procedures have been performed according to international clinical standards with 700µm Embozene particles as described before.^[^ ^65^ ^]^ All patient examinations were approved by the internal ethics committee (internal reference 469/21 S‐KH).

### MR Measurement Set Up

In vitro and EL4 lymphoma tumor progression MR measurements were performed using a 7 T small animal scanner equipped with Agilent (Santa Clara, CA, USA) Discovery MR901 magnet and gradient system and Bruker (Billerica, MA, USA) AVANCE III HD electronics and using ParaVision 6.0.1 software. For cell suspension experiments, spectra were measured with a ^1^H solenoid coil with a 10 mm inner diameter (RAPID Biomedical, Rimpar, Germany). For animal experiments, a dual‐tuned ^1^H/^13^C birdcage resonator with a 31 mm inner diameter (RAPID Biomedical, Rimpar, Germany) was used. This coil allowed proton imaging, shimming, frequency calibration, and ^13^C signal excitation and reception. The animals’ lower backs, where tumors were located, were covered with Carbopol 980 gel (Caesar & Loretz GmbH, Germany) to improve B0 shim uniformity (indicated by * in Figure 3). A urea tube for ^13^C frequency and transmit power calibration was placed near the target region. MR measurements of Colo205 colorectal and EL4 lymphoma tumors before and after treatment were acquired using a 9.4 T small animal scanner and 4 cm inner diameter T/R Millipede coil (Agilent, Palo Alto, CA).

The cell sample and animal rectal temperatures were monitored with a temperature probe and the MR‐compatible, Model 10l monitoring and gating system (SA Instruments, Inc., Stony Brook, USA). For in vitro experiments, cell suspensions were positioned at the scanning position and left for 15 min to reach room temperature of 16–18 °C before measurements started. For in vivo experiments, warm air was blown through the magnet bore by with a Pet Dryer Model B‐8 (XPower, City of Industry, CA, USA) to keep animals’ temperature within the range of 37–39 °C. Breathing rate was kept at 50–70 breaths min^−1^ and monitored with an ECG Trigger Unit (Rapid Biomedical, Rimpar, Germany).

Clinical MR measurements were performed using a 3 T Biograph mMR from Siemens and a ^1^H body matrix coil from Siemens.

### In Vitro MR Parameters

The PGSE sequence parameters were: TE: 19.7 ms, TR: 4000 ms, b: 59; 91; 191; 261; 361; 491; 711; 911; 1211; 1391; 1511; 2011; 2401 s mm^−^^2^, averages: 1, diffusion gradient separation Δ: 10 ms; diffusion gradient duration δ: 4 ms, acquisition time: 52 s.

FEXSY data were collected with TE_f_: 21.8 ms, TE: 21.7 ms, TR: 4000 ms, b: 59; 91; 191; 261; 361; 491; 711; 911; 1211; 1391; 1511; 2011; 2401 s mm^−^^2^, averages: 1, spoiler gradient: 80 mT m^−1^ and 2 ms, and crusher gradient: 80 mT m^−1^ and 1 ms, diffusion gradient separation Δ and Δ_f_: 10 ms; diffusion gradient duration δ and δ_f_: 4 ms. The calculation of one AXR value required two sets of measurements. One set with minimal b_f_: 11 s mm^−^^2^ and t_m_: 4.5 ms, and one set with b_f_: 2044 s mm^−^^2^ and t_m_: 4.5; 14.5; 24.5; 54.5; 114.5; 164.5; 214.5; 264.5; 304.5 ms. The acquisition time per AXR value was 8 min and 40 s.

### EL4 Untreated Mouse Imaging MR Parameters

A multi‐slice T_2_ weighted (T2w) image was used to find the most uniform tumor region (in terms of tumor width and solid tumor) within a 3 mm axial slice, through which the DWI and FEXI slice were placed. The T2w images were acquired with the scanner's stock rapid acquisition with relaxation enhancement (RARE) pulse sequence. The acquisition parameters were: TE: 48 ms, TR: 4000 ms, RARE factor: 8, slice thickness: 1 mm, number of slices: 11, encoding matrix size: 200 × 200, FOV: 2.8 cm × 2.8 cm.

Diffusion‐weighted images (DWI) were acquired with the scanner's stock unipolar spin‐echo diffusion‐weighted sequence with echo‐planar readout. The acquisition parameters were: TE: 21.3 ms, TR: 4000 ms, b: 51; 603 s mm^−^^2^, averages: 1, repetitions: 16, slice thickness: 3 mm, number of slices: 1, encoding matrix size: 32 × 32, FOV: 2.8 cm × 2.8 cm, diffusion gradient separation Δ: 10 ms; diffusion gradient duration δ: 4 ms, Acq. Time: 2 min 8 s.

Filter‐exchange images were acquired with a self‐implemented FEXI sequence that was implemented according to Lasič et al.^[^ ^20^ ^]^ Parameters for the preclinical data acquisition were: TE_f_: 20.8 ms, TE: 21.3 ms, TR: 4000 ms, b: 51; 603 s mm^−^^2^, averages: 1, repetitions min/max filter strength: 16/32, slice thickness: 3 mm, number of slices: 1, encoding matrix size: 32 × 32, FOV: 2.8 cm × 2.8 cm, spoiler gradient: 167 mT m^−1^ and 1 ms, and crusher gradient: 118 mT m^−1^ and 1 ms, diffusion gradient separations Δ and Δ_f_: 10 ms; diffusion gradient duration δ and δ_f_: 4 ms. The calculation of one AXR map used two sets of images: one set with minimal b_f_: 11 s mm^−^^2^ and t_m_: 3.5 ms, and one set with b_f_: 1288 s mm^−^^2^ and t_m_: 13.5; 103.5; 203.5; 303.5 ms. The acquisition time per AXR map was 18 min and 32 s.

2D phase‐encoded chemical shift images (CSI) after injection of hyperpolarized [1,4‐^13^C_2_]fumarate was acquired with a sequence with a custom center‐out order of phase encodes. Data of the first animals were measured dynamically by acquiring multiple CSI image repetitions. Acquisition was started before the injection of the HP compound. Parameters for the dynamic CSI scans were: matrix size: 10 × 8, FOV: 3.0 cm × 2.4 cm, slice thickness: 3 mm, repetition time: 53 ms, 40 repetitions. The acquired spectral bandwidth was 2000 Hz with 100 points. Excitation RF pulse was a Shinnar–Le Roux calculated pulse with sharpness: 3, duration: 0.84 ms, excitation bandwidth: 5 kHz, flip angle 5°. For static measurements, the imaging parameters were: matrix size: 16 × 16, FOV: 2.8 cm × 2.8 cm, slice thickness: 3 mm, repetition time: 72 ms. Acquired spectral bandwidth was 1905 Hz with 128 points. Excitation RF pulse was a Shinnar‐Le Roux calculated pulse with sharpness: 3, duration: 0.5 ms, excitation bandwidth: 8.4 kHz, flip angle: 12°. The measurements were started 25 s after end of injection.

Prior to HP injection, RF power was calibrated using a 300 µL tube containing 2m ^13^C_2_ urea and 2mm gadoteric acid (Dotarem, Guerbet, Villepinte, France) and a custom pulse sequence using non‐localized spectral acquisition and incremented RF transmit powers, which was analyzed in MatLab.^[^ ^66^ ^]^

### Treated EL4 and Colo205 Tumors MR Parameters

The MR data was acquired according to the in vivo protocol previously described by Schilling et al.^[^ ^25^ ^]^

### Human Patient MR Parameters

T2w images were acquired with a turbo spin echo sequence. The acquisition parameters were: TE: 82 ms, TR: 5350 ms, turbo factor: 15, slice thickness: 2.5 mm, number of slices: 5, encoding matrix size: 320 × 272, FOV: 32 cm × 32 cm.

DWI were acquired using the scanner's stock unipolar spin‐echo diffusion‐weighted sequence with echo‐planar readout. The acquisition parameters were: TE: 81 ms, TR: 6600 ms, b: 0; 500 s mm^−^^2^, averages: 1, repetitions: 1, slice thickness: 10 mm, number of slices: 1, encoding matrix size: 130 × 130, FOV: 32 cm × 32 cm, Acq. Time: 1 min 8 s.

A FEXI sequence that was developed according to Lasič et al.^[^ ^23^ ^]^ was used with: TE_f_: 42.5 ms, TE: 72 ms, TR: 4000 ms, b: 0; 500 s mm^−^^2^, averages: 1, repetitions at min and max filter strengths: 10 and 10, slice thickness: 10 mm, number of slices: 1, encoding matrix size: 90 × 90, FOV: 32 cm × 32 cm, spoiler gradient: 34 mT m^−1^ and 2.5 ms, and crusher gradient: 32 mT m^−1^ and 1 ms. The calculation of one AXR map used two sets of images: one set with minimal b_f_: 0 s mm^−^^2^ and t_m_: 5 ms, and one set with b_f_: 700 s mm^−^^2^ and t_m_: 5; 10; 30; 50; 70; 100; 150; 200 ms. The acquisition time per AXR map was 20 min and 40 s.

All data acquisitions of the same cell suspension, animal, or patient were performed consecutively and without the subject being moved. The spectra acquisition scheme was PGSE, FEXSY with minimum b_f_, and FEXSY with maximum b_f_. This scheme was repeated three times for each cell suspension. The imaging acquisition scheme was DWI, FEXI with minimum b_f_, and FEXI with maximum b_f_. Then, in the animal experiments, CSI was performed. All imaging modalities used axial slice orientation.

### Hyperpolarization of Fumarate

For the HP process, 3.9 m [1,4‐^13^C_2_]pyruvate (34 ± 3.0 mg) supplemented with 19 mm OX063 trityl radical and 0.6 mm Dotarem were polarized with a HyperSense (Oxford Instruments, Abingdon, United Kingdom) for ≈90 min at 1.2 K using a microwave frequency of 94.155 GHz and 100 mW power. The sample was dissolved in a solution pressurized to 10 bar and heated to 180 °C containing 2.5 ± 0.3 mL 80 mm Tris buffer supplemented with 80 mm sodium hydroxide (NaOH) and 0.1 g L^−1^ sodium ethylenediaminetetraacetic acid (EDTA). Finally, the solution for injection contained an average fumarate concentration of 80 ± 2.9 mm at pH 7.5 ± 0.3. The polarization level was ≈20% (measured at B_0_ = 1 T, Spinsolve Carbon, Magritek, Aachen, Germany).^[^ ^67^ ^]^ The hyperpolarized solution was rapidly transferred to the preclinical 7T MR scanner and injected 20 s after dissolution.

### In Vitro MR Data Analysis

The Stejskal–Tanner equation (similar to Equation 3) was fitted to the water spectral peak intensities for PGSE data. The data fitting was performed by iterating over different ADC values to minimize the residuals of a least squares regression, with independent variable e^−biADC^ (b_i_ is the diffusion‐weighting factor), dependent variable S_i,_ and regression slope S_0_.

For FEXSY data, Equation (3) was fitted to the water spectral peak intensities as mentioned before, separately for each filter strength (b_f_) and mixing time (t_m_). This resulted in one ADC value for every filter strength (b_f_) and mixing time (t_m_) combination. Then, AXR and filter efficiency (σ) values were calculated by using the Equations (1) and (2) and the MATLAB “fminsearch” function.

### Mice MR Data Analysis

For DWI and FEXI data, the above‐mentioned calculations were performed separately for each voxel to create the ADC and AXR maps. Before any calculation, DWI and FEXI images were filtered with a 2‐D Gaussian smoothing kernel with a standard deviation of 0.6.^[^ ^24^ ^]^ Here, the MATLAB “imgaussfilt” function was used. For each voxel, the signal standard deviation of the repeated measurements with equal b, b_f_ and t_m_ (the last two only in case of FEXI signal) was calculated. Then, the S(b) of the signal decay fitting curve, which was calculated including all repetitions data, was compared with each repetition signal value. The signal values that differed more than 1 and 0.5 times standard deviations were excluded from the ADC and AXR calculation, respectively.

For AXR, ADC and MFR statistical analysis and illustrated maps, only the solid tumor region data were included. Voxels that correspond to solid tumor regions of the EL4 lymphomas were considered those with ADC lower than 12 × 10^−4^ mm^2^ s^−1^ (Figure 3). Furthermore, voxels where the filter efficiency was not between 0 and 1 were excluded from both statistical analysis and illustrated maps of AXR.

MatLab 2021a (The MathWorks, Natick, MA, USA) was used for ADC and AXR values calculations. To compare the variation between in vivo AXR, σ, ADC, and MFR values across all solid tumor regions, the interquartile range (IQR) divided by the median was used.

CSI data was analyzed using Python 3.10. The dynamically acquired CSI data were averaged over repetitions before spectral fitting was performed. The 3D‐data (x‐y‐spectral) (isotropic (3 mm)^3^ voxels) was spatially interpolated to match the resolution of the AXR images (0.875 × 0.875 × 3.0 mm^3^) using cubic interpolation. A per‐voxel fit of the time‐domain data was performed by iteratively minimizing the sum‐of‐squares difference of the measured data and the modeled free induction decay (FID) data, while adjusting model peaks’ frequency, T2^*^, phase, and magnitude for three signal components corresponding to malate (two components) and fumarate (one component). The resulting FID amplitudes of fumarate (F) and malate (M) were divided to get the malate‐to‐fumarate ratio MFR: MFR = M/F. Voxels where the FID amplitudes F of fumarate with a signal‐to‐noise ratio (SNR) less than 0.6 were excluded from further analysis. SNR was determined by calculating, for each voxel, the standard deviation (std) of the real and imaginary components of the time‐domain noisy signals, and then calculating the mean std of voxels within the signal‐void region‐of‐interest (ROI), and then SNR = F/std.

Extraction of the tumor data of CSI, DWI, and FEXI was done by drawing tumor‐ROIs on the high‐resolution anatomical images. CSI, DWI, and FEXI voxels were considered if a voxel of the respective modality was covered by more than 50% ROI.

### Human Patients MR Data Analysis

The ADC and AXR maps of uterine fibroids were calculated similarly to the EL4 lymphomas ADC and AXR maps. Here, signal values that differed more than 1 standard deviation were excluded from the AXR calculation. From the AXR maps after embolization, 4 (uterine fibroid #1) and 2 (uterine fibroid #2) AXR voxel values that were above 70 were considered out of the expected AXR range and were excluded. Furthermore, uterine fibroids’ AXR and ADC maps were filtered with 2D Gaussian smoothing kernel with a standard deviation of 0.6.

### Flow Cytometry

In apoptotic, necrotic and necroptotic cell samples, the fractions of cells with intact vs compromised membranes were determined by flow cytometry using double staining with annexin V and propidium iodide (PI). Annexin V is a protein that binds with high affinity to phosphatidylserine and was used conjugated with the fluorescent allophycocyanin (APC). PI is a fluorescent dye that binds to DNA. The membrane of healthy cells excludes both dyes and thus phosphatidylserine, which is located on the intracellular leaflet of the plasma membrane, and the nuclei are inaccessible to Annexin V and PI. Consequently, healthy cells appear negative to both stains. However, during early stages of necrosis and apoptosis, the plasma membrane undergoes structural alterations, including the translocation of phosphatidylserine from the inner to the outer plasma membrane leaflet. Thus, Annexin V can access the phosphatidylserine, while PI cannot permeate the cells.^[^ ^68^, ^69^ ^]^ During later stages of cell death, the plasma membrane loses its integrity, allowing annexin V and PI to enter the cell; the cells appear positive for both stains.

Cells were harvested, rinsed with 3 ml PBS, then rinsed with 3 ml Annexin V binding buffer (10 mm HEPES, 150 mm NaCl, 2 mm CaCl_2_, 1 mm MgCl_2_, 5 mm KCl_2_, 3% FCS), followed by staining with 5 µL APC‐labeled Annexin V (Biolegend, California, USA) per 100 µL of cells for 15 min at room temperature in the dark. 5 µL of PI staining solution (Sigma–Aldrich, Missouri, USA) was added and incubated for 5‐10 min at room temperature, protected from light. Flow analysis was performed on a BD FACS Canto II (BD Bioscience location) and data were analyzed using FlowJo version 10.1r7 software (TreeStar Inc., Ashland, USA).^[^ ^70^, ^71^ ^]^

### Scanning Electron Microscopy

Necrotic cells were imaged by scanning electron microscopy (Sigma 300 VP, Carl Zeiss AG, Germany). After isopropanol treatment, cells were washed and diluted in PBS (100 cells µL^−1^). Then, 1 µL of cell suspension was positioned on an Al‐glass slide and let dry before it was scanned (Scanning parameters: SE2 detector, working voltage: 10 kV, working distance: 6.0 mm).

### Histology for Mice without Therapy

Tumor cellularity was validated with histological analysis after hematoxylin and eosin (H&E) staining. After animals were sacrificed, the subcutaneous EL4 lymphomas were removed and cut in the same plane as the MR images. The cut position was decided by using the anatomical information of the T2w MRI. Directly after, the two EL4 tumor parts were changed into formalin and fixed for more than 48 h. Then, 3 µm slices from both sides of the cut were stained with hematoxylin and eosin. On the top of one of the slices of each animal, a grid with a 0.5 mm side length was drawn, and the percentage of necrotic cells was estimated visually for every square. Then, the percentages were entered into a MatLab matrix to create the heat maps.

### Histology for Mice with Therapy

Tumors were excised and proceeded for paraffin embedding. 5 µm sections were made. Tumor sections were stained for cleaved caspase‐3, where a rabbit monoclonal anti‐CC3 antibody (cat. 9664, Cell Signaling Technology Inc,Danvers, MA) and a donkey anti‐rabbit secondary biotinylated antibody (Jackson ImmunoResearch Laboratories.West Grove, PA) were used in in a Polymer Refine Kit on an automated Bond platform (Leica Biosystems Ltd, Newcastle, UK); or they were stained using TdT‐mediated dUTP Nick‐End Labeling (TUNEL) using a DeadEnd Colorimetric system kit (Promega Benelux BV, Leiden, The Netherlands). Stained sections were scanned on an Aperio AT2 (Leica Biosystems) at ×20 magnification, with a resolution of 0.5 µm per pixel. All annotations were performed with ImageScope (Leica Biosystems) and the stained surface area was quantified using the algorithm ‘Positive Pixel Count v9’ from Imagescope.

### Mice Fluorescence Imaging

Whole body bioluminescence and fluorescence measurements were acquired using an IVIS 200 series camera (Perkin–Elmer, Waltham, MA). Fluorescence imaging (FLI) measurements were performed with a F‐stop 2 using an ICG filter set. Regions of interest were analyzed using Living Image software (Perkin–Elmer). FLI measurements at various time points (0–24 h) following i.v. injection of C2Am‐750. Regions of interest were analyzed using Living Image software (Perkin‐Elmer).

### Statistical Analysis

Statistical analysis was performed in GraphPad Prism (GraphPad Software, La Jolla, CA).

## Conflict of Interest

P.J.J. is employed at and has stock ownership from Cycuria Therapeutics. He also received honoraria from Novartis, Bristo‐Mysers Squibb, Astra Zeneca, Bayer, Boehringer Ingelheim Austria, Pfizer, SERVIER, Roche/Genentech, MSD, Pierre Fabre, Janssen, Merck KGa, Sanofi/Aventis, Ipsen, Amgen, Pierre Fabre, Eli Lilly, Janssen and MSD Oncology.

## Author Contributions

A.K., K.M.B., and F.S. conceived and designed the study. A.K., L.N., U.H., I.B., B.X., and F.S. performed data acquisition. G.J.T., F.H.A., U.H., M.S., and S.S. assisted with data acquisition. A.K., L. N., J.R., and T.A.K. performed MRI sequence development. G.J.T. and J.R. assisted with method development. A.K., L.N., S.B., T.M., and K.S. performed data analysis. G.J.T. assisted with data analysis. C.L., J.N., and P.P. performed patient recruiting. M.S. and B.E interacted with patients. All co‐authors read and edited the manuscript draft. A.K., G.J.T., L.N., U.H., B.X., K.M.B., and F.S. wrote the initial manuscript draft.

## Supporting information



Supporting Information

## Data Availability

The data that support the findings of this study are available from the corresponding author upon reasonable request.

## References

[1] S. Amin , O. F. Bathe , BMC Cancer 2016, 16, 850. 27814715 10.1186/s12885-016-2886-9 PMC5097425

[2] F. S. Collins , H. Varmus , N. Engl. J. Med. 2015, 372, 793. 25635347 10.1056/NEJMp1500523 PMC5101938

[3] E. A. Eisenhauer , P. Therasse , J. Bogaerts , L. H. Schwartz , D. Sargent , R. Ford , J. Dancey , S. Arbuck , S. Gwyther , M. Mooney , L. Rubinstein , L. Shankar , L. Dodd , R. Kaplan , D. Lacombe , J. Verweij , Eur. J. Cancer 2009, 45, 228. 19097774 10.1016/j.ejca.2008.10.026

[4] S. Stroobants , J. Goeminne , M. Seegers , S. Dimitrijevic , P. Dupont , J. Nuyts , M. Martens , B. van den Borne , P. Cole , R. Sciot , H. Dumez , S. Silberman , L. Mortelmans , A. van Oosterom , Eur. J. Cancer 2003, 39, 2012. 12957455 10.1016/s0959-8049(03)00073-x

[5] D. A. Torigian , S. S. Huang , M. Houseni , A. Alavi , CA Cancer J. Clin. 2007, 57, 206. 17626118 10.3322/canjclin.57.4.206

[6] C. J. Galban , B. A. Hoff , T. L. Chenevert , B. D. Ross , NMR Biomed. 2017, 30, 3458. 10.1002/nbm.3458 PMC4947029 26773848

[7] T. F. Nunes , D. Szejnfeld , J. Szejnfeld , C. E. Kater , S. Faintuch , C. H. M. Castro , S. M. Goldman , AJR Am. J. Roentgenol. 2016, 207, 804. 27490448 10.2214/AJR.16.16207

[8] B. A. Moffat , D. E. Hall , J. Stojanovska , P. J. McConville , J. B. Moody , T. L. Chenevert , A. Rehemtulla , B. D. Ross , Magn. Reson. Mater. Phys. Biol. Med. 2004, 17, 249. 10.1007/s10334-004-0079-z 15580371

[9] P. Weerasinghe , L. M. Buja , Exp. Mol. Pathol. 2012, 93, 302. 23036471 10.1016/j.yexmp.2012.09.018

[10] Y. Zhang , X. Chen , C. Gueydan , J. Han , Cell Res. 2018, 28, 9. 29076500 10.1038/cr.2017.133 PMC5752838

[11] Z. Cai , S. Jitkaew , J. Zhao , H.‐C. Chiang , S. Choksi , J. Liu , Y. Ward , L.‐G. Wu , Z.‐G. Liu , Nat. Cell Biol. 2014, 16, 55. 24316671 10.1038/ncb2883 PMC8369836

[12] X. Chen , W. Li , J. Ren , D. Huang , W.‐T. He , Y. Song , C. Yang , W. Li , X. Zheng , P. Chen , J. Han , Cell Res. 2014, 24, 105. 24366341 10.1038/cr.2013.171 PMC3879712

[13] H. Wang , L. Sun , L. Su , J. Rizo , L. Liu , L.‐F. Wang , F.‐S. Wang , X. Wang , Mol. Cell 2014, 54, 133. 24703947 10.1016/j.molcel.2014.03.003

[14] R. A. Aglietti , A. Estevez , A. Gupta , M. G. Ramirez , P. S. Liu , N. Kayagaki , C. Ciferri , V. M. Dixit , E. C. Dueber , Proc. Natl. Acad. Sci. 2016, 113, 7858. 27339137 10.1073/pnas.1607769113 PMC4948338

[15] J. Ding , K. Wang , W. Liu , Y. She , Q. Sun , J. Shi , H. Sun , D.‐C. Wang , F. Shao , Nature 2016, 535, 111. 27281216 10.1038/nature18590

[16] X. Liu , Z. Zhang , J. Ruan , Y. Pan , V. G. Magupalli , H. Wu , J. Lieberman , Nature 2016, 535, 153. 27383986 10.1038/nature18629 PMC5539988

[17] T. V. Berghe , N. Vanlangenakker , E. Parthoens , W. Deckers , M. Devos , N. Festjens , C. J. Guerin , U. T. Brunk , W. Declercq , P. Vandenabeele , Cell Death Differ. 2010, 17, 922. 20010783 10.1038/cdd.2009.184

[18] C. Rogers , T. Fernandes‐Alnemri , L. Mayes , D. Alnemri , G. Cingolani , E. S. Alnemri , Nat. Commun. 2017, 8, 14128. 28045099 10.1038/ncomms14128 PMC5216131

[19] P. T. Callaghan , I. Furo , J. Chem. Phys. 2004, 120, 4032. 15268569 10.1063/1.1642604

[20] Y. Kharbanda , M. Urbańczyk , V. V. Zhivonitko , S. Mailhiot , M. I. Kettunen , T. V. Sensitive , Angew. Chem. Int. Ed. 2022, 61, 202203957. 10.1002/anie.202203957 PMC9400989 35499690

[21] N. H. Williamson , R. Ravin , T. X. Cai , M. Falgairolle , M. J. O'Donovan , P. J. Basser , PNAS Nexus 2023, 2, pgad056. 36970182 10.1093/pnasnexus/pgad056 PMC10032361

[22] I. Aslund , A. Nowacka , M. Nilsson , D. Topgaard , J. Magn. Reson. 2009, 200, 291. 19647458 10.1016/j.jmr.2009.07.015

[23] S. Lasic , M. Nilsson , J. Latt , F. Stahlberg , D. Topgaard , Magn. Reson. Med. 2011, 66, 356. 21446037 10.1002/mrm.22782

[24] M. Nilsson , J. Lätt , D. van Westen , S. Brockstedt , S. Lasic , F. Ståhlberg , D. Topgaard , Magn. Reson. Med. 2013, 69, 1572. 10.1002/mrm.24395 22837019

[25] F. Schilling , S. Ros , D.‐E. Hu , P. D'Santos , S. McGuire , R. Mair , A. J. Wright , E. Mannion , R. J. M. Franklin , A. A. Neves , K. M. Brindle , Nat. Biotechnol. 2017, 35, 75. 27918546 10.1038/nbt.3714 PMC5230773

[26] M. Schillmaier , A. Kaika , G. J. Topping , R. Braren , F. Schilling , MAGMA 2023, 36, 957. 37436611 10.1007/s10334-023-01107-w PMC10667135

[27] M. Schillmaier , A. Kaika , F. Schilling , Advanced Diffusion Encoding Methods in MRI, Vol. 24, The Royal Society of Chemistry, London, UK 2020, pp. 154–185

[28] B. Lampinen , F. Szczepankiewicz , D. van Westen , E. Englund , P. CS , J. Latt , F. Ståhlberg , M. Nilsson , Magn. Reson. Med. 2017, 77, 1104. 26968557 10.1002/mrm.26195 PMC5324642

[29] D. A. Dominguez , L. W. Thornblade , Ann. Surg. Oncol. 2022, 29, 2752. 35235086 10.1245/s10434-022-11481-9

[30] M. J. Pollheimer , P. Kornprat , R. A. Lindtner , L. Harbaum , A. Schlemmer , P. Rehak , C. Langner , Hum. Pathol. 2010, 41, 1749. 20869096 10.1016/j.humpath.2010.04.018

[31] P. Borchmann , J. Ferdinandus , G. Schneider , A. Moccia , R. Greil , M. Hertzberg , V. Schaub , A. Hüttmann , F. Keil , J. Dierlamm , M. Hänel , U. Novak , J. Meissner , A. Zimmermann , S. Mathas , J. M. Zijlstra , A. Fosså , A. Viardot , B. Hertenstein , S. Martin , P. Giri , S. Scholl , M. S. Topp , W. Jung , V. Vucinic , H.‐J. Beck , A. Kerkhoff , B. Unger , A. Rank , R. Schroers , et al., Lancet 2024, 404, 341. 38971175 10.1016/S0140-6736(24)01315-1

[32] F. A. Gallagher , M. I. Kettunen , D.‐E. Hu , P. R. Jensen , R. I. 'T. Zandt , M. Karlsson , A. Gisselsson , S. K. Nelson , T. H. Witney , S. E. Bohndiek , G. Hansson , T. Peitersen , M. H. Lerche , K. M. Brindle , Proc. Natl. Acad. Sci. USA 2009, 106, 19801. 19903889 10.1073/pnas.0911447106 PMC2785247

[33] F. Hesse , V. Somai , F. Kreis , F. Bulat , A. J. Wright , K. M. Brindle , Proc. Natl. Acad. Sci. USA 2021, 118, e2014631118. 33727417 10.1073/pnas.2014631118 PMC8000230

[34] F. Hesse , A. J. Wright , F. Bulat , V. Somai , F. Kreis , K. M. Brindle , Magn. Reson. Med. 2022, 88, 2014. 35816502 10.1002/mrm.29379 PMC9545469

[35] F. Hesse , A. J. Wright , V. Somai , F. Bulat , F. Kreis , K. M. Brindle , Cancer Res. 2022, 82, 3622. 35972377 10.1158/0008-5472.CAN-22-0101 PMC9530651

[36] S. E. Bohndiek , M. I. Kettunen , D. Hu , T. H. Witney , B. W. C. Kennedy , F. A. Gallagher , K. M. Brindle , Mol. Cancer Ther. 2010, 9, 3278. 21159611 10.1158/1535-7163.MCT-10-0706 PMC3003424

[37] A. Kaika , G. J. Topping , L. Nagel , F. Schilling , NMR Biomed. 2024, 37, 5202. 10.1002/nbm.5202 38953779

[38] J. Balvan , A. Krizova , J. Gumulec , M. Raudenska , Z. Sladek , M. Sedlackova , R. Kizek , R. Chmelik , M. Masarik , PLoS One 2015, 10, 0121674. 10.1371/journal.pone.0121674 PMC4372376 25803711

[39] A. Montecucco , F. Zanetta , G. Biamonti , EXCLI J. 2015, 14, 95. 26600742 10.17179/excli2015-561 PMC4652635

[40] V. Grinkevitch , M. Wappett , N. Crawford , S. Price , A. Lees , C. McCann , K. McAllister , J. Prehn , J. Young , J. Bateson , L. Gallagher , M. Michaut , V. Iyer , A. Chatzipli , S. Barthorpe , D. Ciznadija , I. Sloma , A. Wesa , D. A. Tice , L. Wessels , M. Garnett , D. B. Longley , U. McDermott , S. S. McDade , Mol. Cancer Ther. 2022, 21, 594. 35086954 10.1158/1535-7163.MCT-21-0532 PMC7612587

[41] D. Dao , S. J. Kang , M. Midia , Diagn. Interv. Radiol. 2019, 25, 157. 30774092 10.5152/dir.2019.18294 PMC6411268

[42] S. Lasic , S. Oredsson , S. C. Partridge , L. H. Saal , D. Topgaard , M. Nilsson , K. Bryskhe , NMR Biomed. 2016, 29, 631. 26929050 10.1002/nbm.3504 PMC4833667

[43] A.‐S. GK , Biochem. Soc. Trans. 2021, 49, 793. 33843978 10.1042/BST20200696 PMC8106503

[44] I. K. H. Poon , C. D. Lucas , A. G. Rossi , K. S. Ravichandran , Nat. Rev. Immunol. 2014, 14, 166. 24481336 10.1038/nri3607 PMC4040260

[45] M. Sachet , Y. Y. Liang , R. Oehler , Apoptosis 2017, 22, 1189. 28861714 10.1007/s10495-017-1413-z PMC5630647

[46] T. Lee , T. Lau , I. Ng , Cancer Chemother. Pharmacol. 2002, 49, 78. 11855756 10.1007/s00280-001-0376-4

[47] B. A. Carneiro , W. S. El‐Deiry , Nat. Rev. Clin. Oncol. 2020, 17, 395. 32203277 10.1038/s41571-020-0341-y PMC8211386

[48] A. D. Garg , E. Romano , N. Rufo , P. Agostinis , Cell Death Differ. 2016, 23, 938. 26891691 10.1038/cdd.2016.5 PMC4987738

[49] I. K. H. Poon , M. D. Hulett , C. R. Parish , Cell Death Differ. 2010, 17, 381. 20019744 10.1038/cdd.2009.195

[50] Z. Liu , D. Jiao , Cell Stress 2020, 4, 1. 10.15698/cst2020.01.208 PMC6946014 31922095

[51] X. Tong , R. Tang , M. Xiao , J. Xu , W. Wang , B. Zhang , J. Liu , X. Yu , S. Shi , J. Hematol. Oncol. 2022, 15, 174. 36482419 10.1186/s13045-022-01392-3 PMC9733270

[52] D. R. Miller , S. D. Cramer , A. Thorburn , Int. Rev. Cell Mol. Biol. 2020, 352, 159. 32334815 10.1016/bs.ircmb.2019.12.004 PMC8185908

[53] Y. Y. Sabri , N. M. Ewis , H. E. H. Zawam , M. A. Khairy , Egypt. J. Radiol. Nucl. Med. 2021, 52, 215.

[54] C. Messina , R. Bignone , A. Bruno , A. Bruno , F. Bruno , M. Calandri , D. Caruso , P. Coppolino , R. De Robertis , F. Gentili , I. Grazzini , R. Natella , P. Scalise , A. Barile , R. Grassi , D. Albano , Cancers 2020, 12, 1493. 32521645 10.3390/cancers12061493 PMC7352852

[55] T. Hauser , M. Essig , A. Jensen , F. B. Laun , M. Münter , K. H. Maier‐Hein , B. Stieltjes , Eur. J. Radiol. 2014, 83, 783. 24631600 10.1016/j.ejrad.2014.02.013

[56] N. Faye , O. Pellerin , R. Thiam , F. Chammings , M. Brisa , E. Marques , C. A. Cuénod , M. Sapoval , L. S. Fournier , Magn. Reson. Med. 2013, 70, 1739. 23440651 10.1002/mrm.24624

[57] Y. Gu , C. Zhou , Z. Piao , H. Yuan , H. Jiang , H. Wei , Y. Zhou , G. Nan , X. Ji , Front. Neurosci. 2022, 16, 988283. 36061592 10.3389/fnins.2022.988283 PMC9434007

[58] M. G. Lansberg , V. N. Thijs , M. W. O'Brien , J. O. Ali , A. J. de Crespigny , D. C. Tong , M. E. Moseley , G. W. Albers , AJNR Am. J. Neuroradiol. 2001, 22, 637. 11290470 PMC7976036

[59] K. Y. Loh , Z. Wang , P. Liao , Reviews of Physiology, Biochemistry and Pharmacology, Vol. 176, Springer International Publishing, Cham, Switzerland 2019, pp. 37–64. 30515590 10.1007/112_2018_13

[60] S. Wei , B. Chen , S. W. Low , C. P. Poore , Y. Gao , B. Nilius , P. Liao , Mol. Neurobiol. 2023, 60, 5931. 37380823 10.1007/s12035-023-03453-1 PMC10471688

[61] M. Vu , N. Kassouf , R. Ofili , T. Lund , C. Bell , S. Appiah , Int. J. Oncol. 2020, 57, 113. 32377726 10.3892/ijo.2020.5052 PMC7252449

[62] M. Patra , E. Salonen , E. Terama , I. Vattulainen , R. Faller , B. W. Lee , J. Holopainen , M. Karttunen , Biophys. J. 2006, 90, 1121. 16326895 10.1529/biophysj.105.062364 PMC1367264

[63] G. Brumatti , C. Ma , N. Lalaoui , N.‐Y. Nguyen , M. Navarro , M. C. Tanzer , J. Richmond , M. Ghisi , J. M. Salmon , N. Silke , G. Pomilio , S. P. Glaser , E. de Valle , R. Gugasyan , M. A. Gurthridge , S. M. Condon , R. W. Johnstone , R. Lock , G. Salvesen , A. Wei , D. L. Vaux , P. G. Ekert , J. Silke , Sci. Transl. Med. 2016, 8, 339ra69. 10.1126/scitranslmed.aad3099 27194727

[64] A. A. Neves , B. Xie , S. Fawcett , I. S. Alam , T. H. Witney , M. M. de Backer , J. Summers , W. Hughes , S. McGuire , D. Soloviev , J. Miller , W. J. Howat , D.‐E. Hu , T. B. Rodrigues , D. Y. Lewis , K. M. Brindle , J. Nucl. Med. 2017, 58, 881. 28209913 10.2967/jnumed.116.183004

[65] H. Van Overhagen , J. A. Reekers , Cardiovasc. Intervent. Radiol. 2015, 38, 536. 25465064 10.1007/s00270-014-1031-x

[66] G. J. Topping , I. Heid , M. Trajkovic‐Arsic , L. Kritzner , M. Grashei , C. Hundshammer , M. Aigner , J. G. Skinner , R. Braren , F. Schilling , Biomedicines 2021, 9, 121. 33513763 10.3390/biomedicines9020121 PMC7911979

[67] C. Hundshammer , S. Duwel , D. Ruseckas , G. Topping , P. Dzien , C. Muller , B. Feuerecker , J. B. Hövener , A. Haase , M. Schwaiger , S. J. Glaser , F. Schilling , Sensors 2018, 18, 600. 29462891 10.3390/s18020600 PMC5856118

[68] M. Van Engeland , L. J. W. Nieland , F. C. S. Ramaekers , B. Schutte , C. P. M. Reutelingsperger , Cytometry 1998, 31, 1. 9450519 10.1002/(sici)1097-0320(19980101)31:1<1::aid-cyto1>3.0.co;2-r

[69] H. Sawai , N. Domae , Biochem. Biophys. Res. Commun. 2011, 411, 569. 21763280 10.1016/j.bbrc.2011.06.186

[70] F. Wallberg , T. Tenev , P. Meier , Cold Spring Harb. Protoc. 2016, 2016, pdbprot087387. 10.1101/pdb.prot087395 26933245

[71] L. C. Crowley , B. J. Marfell , A. P. Scott , N. J. Waterhouse , Cold Spring Harb. Protoc. 2016, 2016, pdbprot087288. 10.1101/pdb.prot087221 27698233

