# Supplementary material for: Early Detection of Cell Death Using Transmembrane Water Exchange Magnetic Resonance Imaging
Source: Adv Sci (Weinh). 2025 Dec 17;13(23):e13317. doi: 10.1002/advs.202513317 (PMC13104136; doi:10.1002/advs.202513317)
Supplement: Supplementary file 1 — Supporting Information [file ADVS-13-e13317-s001.docx]

**Supplementary material**

**Early detection of cell death using transmembrane water exchange magnetic resonance imaging**

Athanasia Kaika, Luca Nagel, Ulrike Höckendorf, Bangwen Xie, Geoffrey J. Topping, Mathias Schillmaier, Irina Beer, Frits H. A. van Heijster, Julian Rauch, Tristan A. Kuder, Sandra Sühnel, Bernd Erber, Christian Lohrmann, Simone Ballke, Thomas Metzler, Katja Steiger, Philipp Jost, Natalia P. Ivleva, Philipp Paprottka, Jonathan Nadjiri, Kevin M. Brindle, Wolfgang A. Weber, and Franz Schilling

**
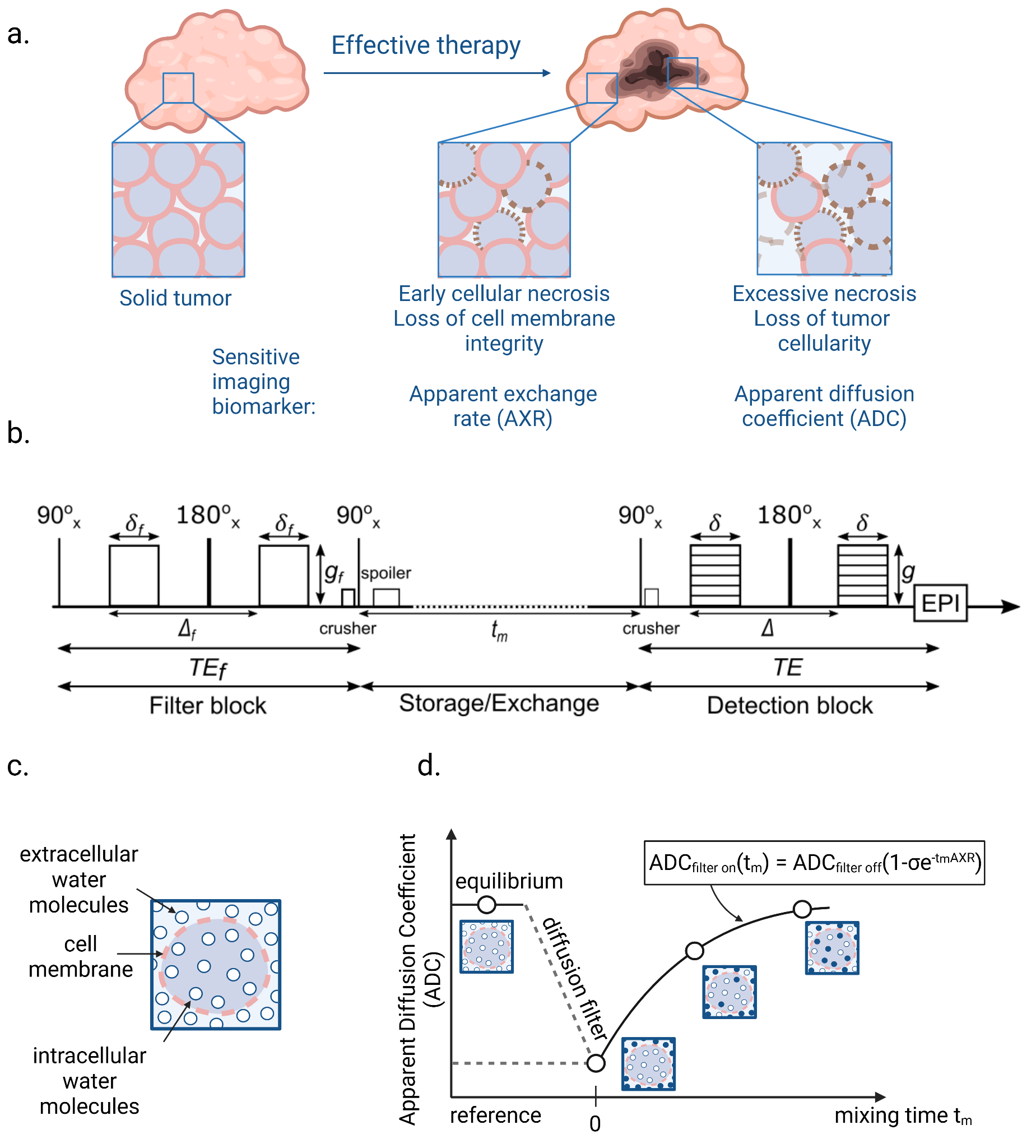
**

**Supplementary Figure S1**

**Schematic representation of cell membrane integrity changes caused by successful treatment of a solid tumour and FEXI sequence that is used to sensitize MR signal to those changes.** a. Depiction of a solid tumour with progressively compromised membrane integrity after the initiation of cell death caused by an effective treatment. Loss of tumour cellularity (excessive necrosis) occurs during the late stages of cell death. The response of a solid tumour to a treatment may be monitored soon after the treatment starts by the apparent exchange rate (AXR), while the apparent diffusion coefficient (ADC) is sensitive to later stages of cell death. b. Pulse sequence diagram of filter-exchange imaging (FEXI). The sequence consists of two diffusion modules (filter and detection blocks) and one storage/exchange time interval with variable mixing times *t_m_*. c. Water molecules diffuse throughout the intra and extracellular space. FEXI uses two diffusion blocks to benefit from the diffusivity difference between intra and extracellular water resulting from water molecules' collisions with the cell membrane and the intracellular structures. d. The first diffusion (filter) block attenuates the fast-diffusing (extracellular) molecules indicated by filled blue circles. Diffusivity is measured by the second (detection) block at different mixing times. The increase of the ADC while increasing the mixing time (t_m_) is caused by the translocation of the initially intracellular water molecules to the fast-diffusing compartment through the cell membrane. The relaxation rate of the ADC back to the ADC value before the diffusion filter application (ADC equilibrium) is the AXR.

**
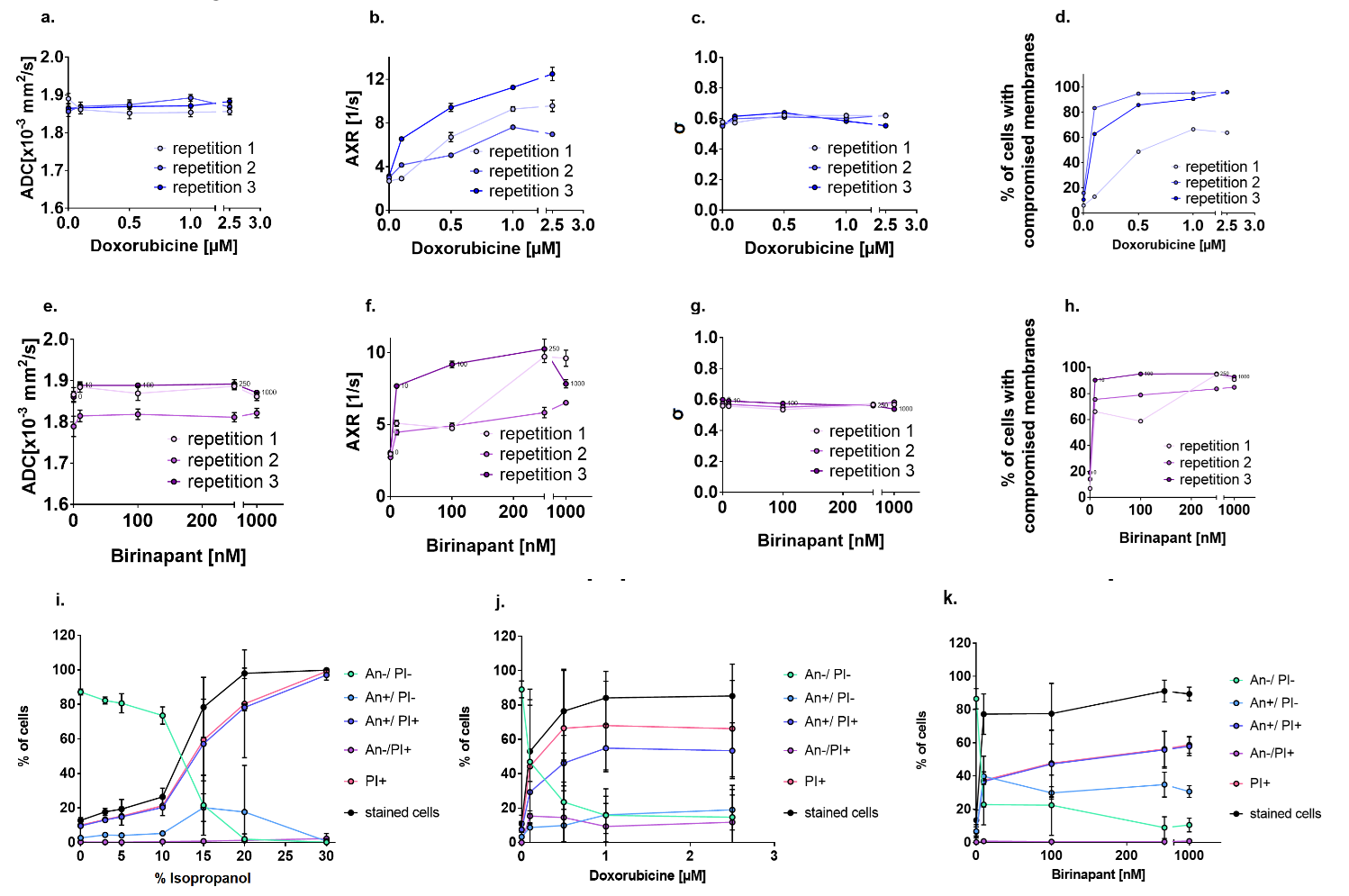
**

**Supplementary Figure S2**

**FEXSY, PGSE, flow cytometry and SEM data of AML cells undergoing apoptosis, necroptosis and necrosis.** a. ADC, b. AXR, c. σ and d. percentage of cells with membranes undergoing structural changes and with compromised integrity (percentage of cells in the gates Q1 + Q2 + Q3) as a function of the doxorubicin concentrations. e. ADC, f. AXR, g. σ and h. percentage of cells with membranes undergoing structural changes and with compromised integrity (percentage of cells in the gates Q1 + Q2 + Q3) as a function of the birinapant concentrations. Each data point corresponds to the mean value, and the whiskers represent one standard deviation above and below the mean of the three consecutive measurements of the same sample. i., j., k. Percentage of: live cells (Q4, Annexin V(-)/PI(-)); early necrotic cells (Q3, Annexin V(+)/PI(-)); late necrotic cells (Q2, Annexin V(+)/PI(+) or Q1, Annexin V(-)/PI(+) or Q2 + Q1, PI(+)) after treatment with varying concentrations of isopropanol, doxorubicin and birinapant. Each data point corresponds to the mean value, and the whiskers represent one standard deviation above and below the mean of the points of the three different samples prepared and measured on different days.


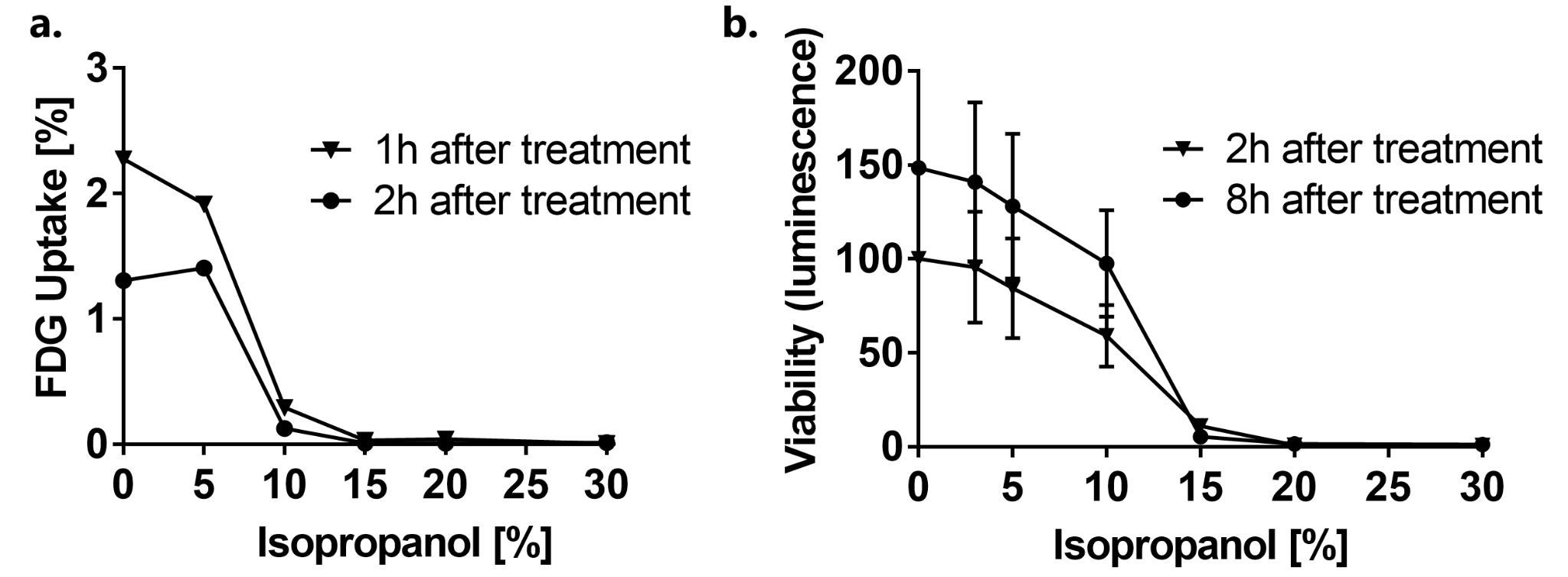


**Supplementary Figure S3**

**Cell viability was tested using FDG Uptake and Luminescence measurements at two different time points.** a. FDG uptake was measured at indicated timepoints for varying isopropanol concentrations. b. Cell viability was detected at indicated time points by RealTime-Glo® MT Cell Viability Assay (Promega) according to the manufacturer’s instructions. Increasing isopropanol concentration reduces both metabolic rate and cell viability.


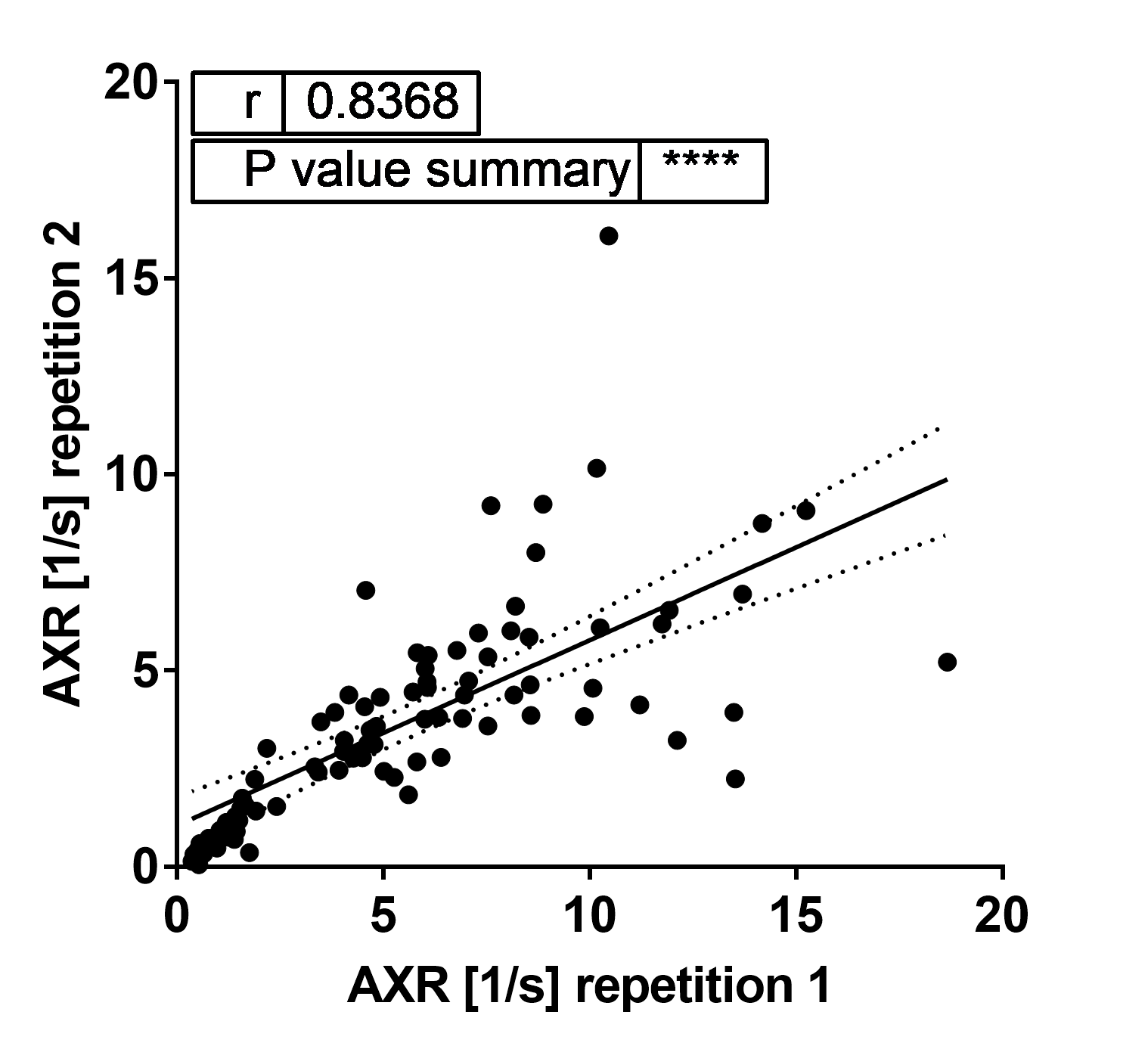


**Supplementary Figure S4**

***In vivo* FEXI repeatability test.** Scatter plot of AXR voxel values the same EL4 lymphoma calculated by two consecutive FEXI acquisitions without repositioning the mouse. Correlation coefficient (r) and P-value was calculated using Spearman correlation; n.s. P > 0.05, * P ≤ 0.05, ** P ≤ 0.01, *** P ≤ 0.001, **** P ≤ 0.0001.


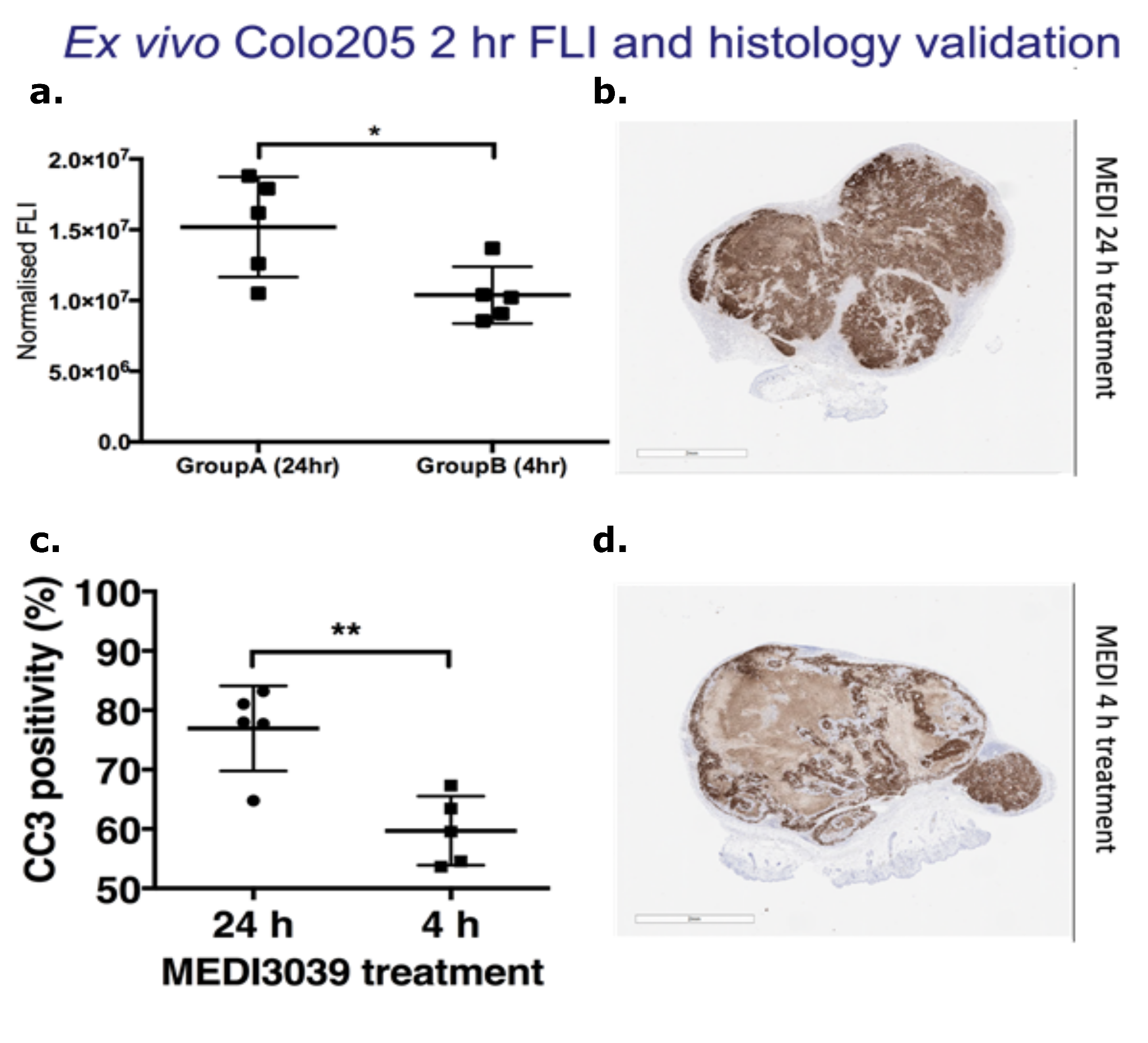


**Supplementary Figure S5**

**Ex vivo validation of Colo205 tumour cell death in FLI and Histology.** MEDI3039-treated Colo205 tumours (24 h vs 4 h) were excised at 2 h post probe injection of C2Am-750. Paraffin embedded tumour sections were scanned for fluorescence imaging. Quantitation of fluorescence intensities in excised tumours from animals injected with C2Am-750 were normalized to tumour weight (**a**). The same sections were followed by histological staining for cleaved caspase-3 (CC3). CC3 positivity percentage was calculated by comparing CC3-positive staining in non-consecutive tumour sections (24 h vs 4 h, four sections per tumour, **c**) and representative scans were shown for visualization of cell death at 24 h and 6 h post-treatment (in brown, **b, d**). * P ≤ 0.05, ** P ≤ 0.01, scale bar = 2 mm.


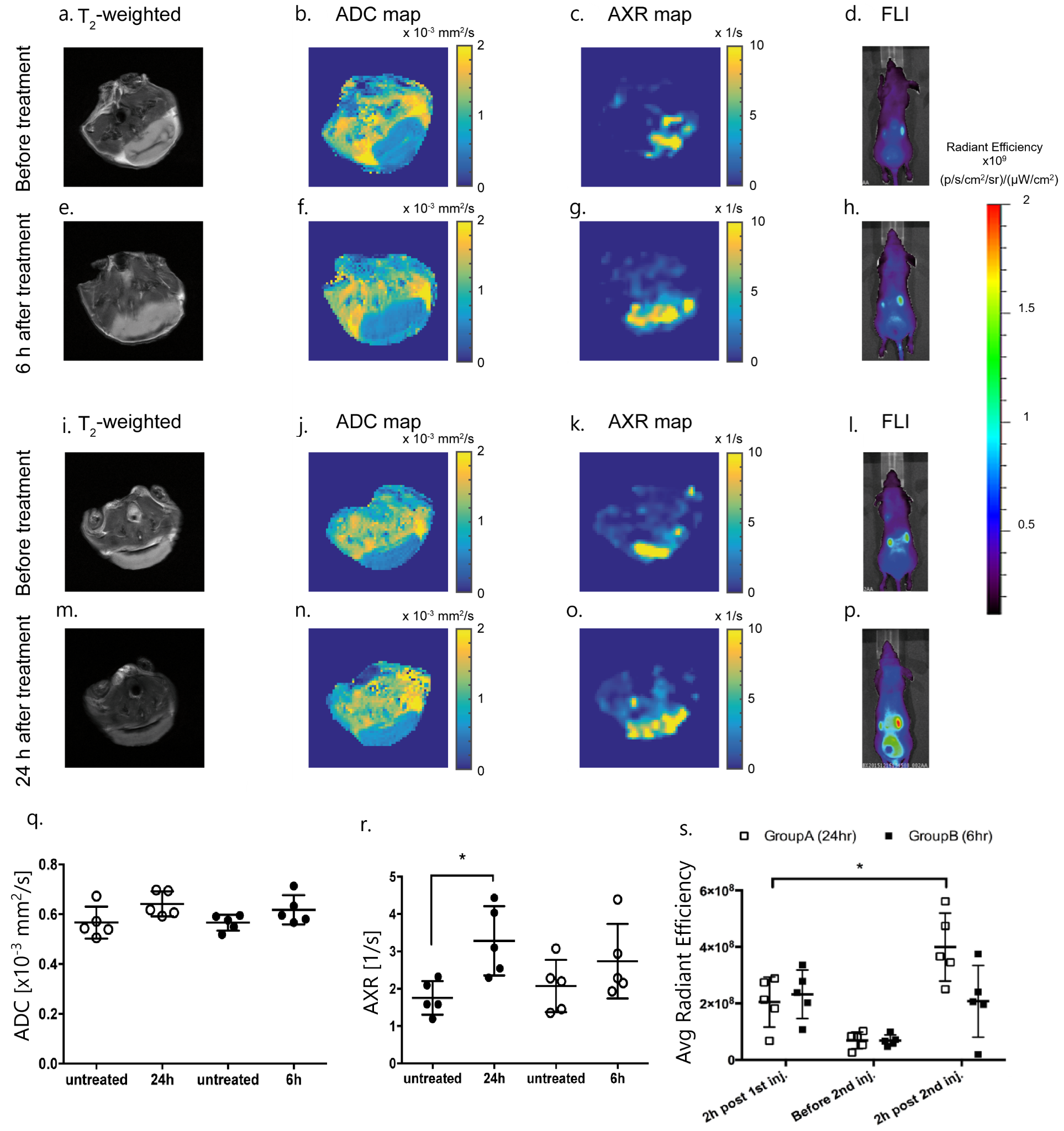


**Supplementary Figure S6**

**Axial MRI (T2-weighted, DWI, FEXI) and fluorescent imaging (FLI) radiant efficiency images** **of mice bearing EL4 lymphomas acquired before and 6 h and 24 h after treatment.** a, e, i, m. Axial T2w, b, f, j, n. ADC, c, u, k, o. AXR, and d, h, l, p. fluorescent radiant efficiency (FLI) images of two mice. q. ADC, r. AXR and s. average radiant efficiency of two groups of five EL4 tumours each. The first injection of C2Am-750 was done before treatment, whereas the second injection was performed after treatment. Points represent single tumour mean values. For each group of five tumours, mean and standard deviation are indicated by horizontal lines and whiskers. Two‐tailed, pairwise Student’s t tests were used, * P ≤ 0.05, ** P ≤ 0.01, *** P ≤ 0.001.


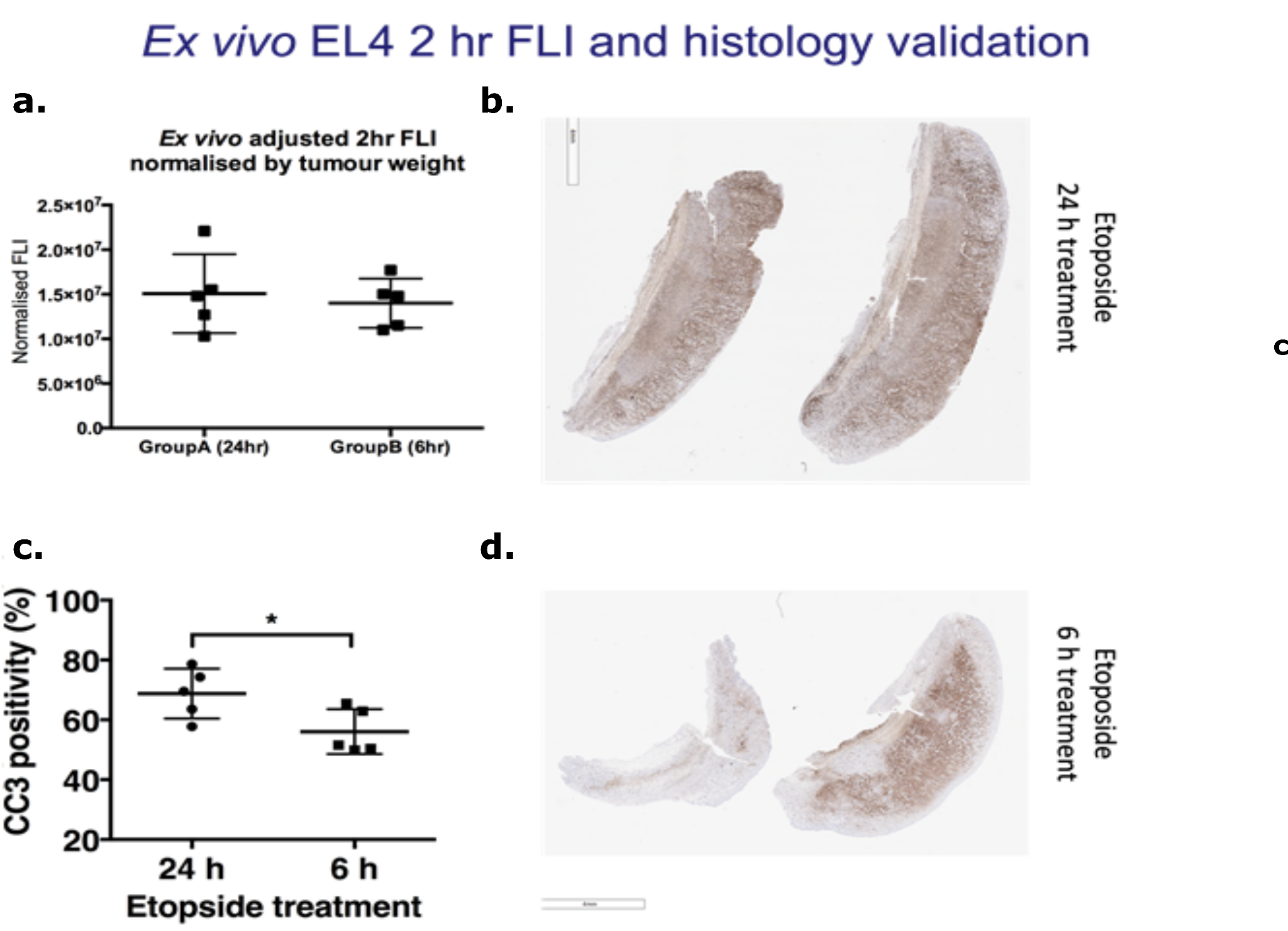


**Supplementary Figure S7**

***Ex vivo* validation of EL4 tumour cell death in FLI and Histology.** MEDI3039-treated EL4 tumours (24 h vs 6 h) were excised at 2 h post probe injection of C2Am-750. Paraffin embedded tumour sections were scanned for fluorescence imaging. Quantitation of fluorescence intensities in excised tumours from animals injected with C2Am-750 were normalized to tumour weight (**a**). The same sections were followed by histological staining for CC3. CC3 positivity percentage was calculated by comparing CC3-positive staining in non-consecutive tumour sections (24 h vs 6 h, four sections per tumour, **c**) and representative scans were shown for visualization of cell death at 24 h and 6 h post-treatment (in brown, **b, d**). * P ≤ 0.05, ** P ≤ 0.01, scale bar = 2 mm.
